# Regulation of epithelial-mesenchymal transition and organoid morphogenesis by a novel TGFβ-TCF7L2 isoform-specific signaling pathway

**DOI:** 10.1038/s41419-020-02905-z

**Published:** 2020-08-25

**Authors:** Kunal Karve, Stuart Netherton, Lili Deng, Azad Bonni, Shirin Bonni

**Affiliations:** 1 Department of Biochemistry and Molecular Biology, Arnie Charbonneau Cancer Institute, Cumming School of Medicine, University of Calgary, Calgary, AB Canada; 2 Department of Neuroscience, Washington University School of Medicine, St. Louis, MO 63110 USA

**Keywords:** Cell biology, Molecular biology

## Abstract

Alternative splicing contributes to diversification of gene function, yet consequences of splicing on functions of specific gene products is poorly understood. The major transcription factor TCF7L2 undergoes alternative splicing but the biological significance of TCF7L2 isoforms has remained largely to be elucidated. Here, we find that the TCF7L2 E-isoforms maintain, whereas the M and S isoforms disrupt morphogenesis of 3D-epithelial cell-derived organoids via regulation of epithelial-mesenchymal transition (EMT). Remarkably, TCF7L2E2 antagonizes, whereas TCF7L2M2/S2 promotes EMT-like effects in epithelial cells induced by transforming growth factor beta (TGFβ) signaling. In addition, we find TGFβ signaling reduces the proportion of TCF7L2E to TCF7L2M/S protein in cells undergoing EMT. We also find that TCF7L2 operates via TGFβ-Smad3 signaling to regulate EMT. Collectively, our findings unveil novel isoform-specific functions for the major transcription factor TCF7L2 and provide novel links between TCF7L2 and TGFβ signaling in the control of EMT-like responses and epithelial tissue morphogenesis.

## Introduction

Protein-encoding genes are limited in number to account for the biological complexity of living organisms^1^. Post-transcriptional mechanisms, including alterative splicing of multi-exonic genes, can lead to increase in the repertoire of cellular proteins, and may contribute to biological diversity. Characterizing the functional implications of distinct splice isoforms of a gene should add insight into the role of alternative splicing in biological versatility. Elucidating the functional significance of different isoforms may also help ascertain how perturbation of expression of mRNA and protein isoforms contributes to disease.

Transcription Factor-7 Like-2 (TCF7L2) is a member of T-cell factors (TCF) family of high mobility group (HMG) box-containing transcription factor^2,3^. TCF7L2 acts in Wnt-β-catenin-signaling-dependent and independent manner to regulate transcription and biological responses including cell proliferation and fate determination^4–9^. TCF7L2 is encoded by the *TCF7L2* gene, with the open reading frame comprising 17 exons^3^. Alternative splicing of exons 4, 13, 14, 15, or 16 in the TCF7L2 pre-mRNA can generate several TCF7L2 mRNA isoforms. In particular, alternative splicing of exons 13–16 generates one of three predominate sets of TCF7L2 isoforms termed extended (E), medium (M), and short (S)^3,6,10–12^. A cysteine-clamp (C-clamp) domain and C-terminal binding protein (CtBP) binding motifs are present C-terminally in the E isoforms, which are absent in the M isoforms, and only a partial C-clamp domain is present in the S isoforms^3,11^. Each the E, M, and S isoforms may also retain or lack certain amino acids, including by splicing in or out of exon 4, thus leading to further complexity^3,13^. The E, M, and S TCF7L2 protein isoforms share exon 1-encoded N-terminal β-catenin binding domain, which is critical for control of the Wnt pathway^3,14^. These TCF7L2 isoforms also share an HMG-box domain and a nuclear localization sequence (NLS) motif, encoded by exons 10–12, which contribute to their ability to control gene expression^3^.

Despite a great deal of interest^3,10,11,15^, the functional significance of TCF7L2 isoforms remains poorly elucidated. TCF7L2 is expressed in epithelial tissues including the mammary glands, skin, and gastrointestinal tract, and may contribute to the maintenance and differentiation of epithelial cells^15–19^. However, TCF7L2 isoform-dependent roles in epithelial cell and tissue maintenance remain to be identified, and are, thus, the focus of this study.

The ability of epithelial cells to transdifferentiate into a mesenchymal phenotype via the epithelial-mesenchymal transition (EMT), is fundamental to the developing organism, and contributes to postnatal mammary gland development and wound healing^20,21^. Epithelial cells undergoing EMT lose their apical–basal polarity and epithelial phenotype and gain fibroblastic-like features, which occur in part due to loss or mislocalization of cell–cell junctional epithelial markers including E-cadherin^22,23^. EMT also leads to increased cell migration and invasion^24^. EMT can be reinitiated in pathological conditions including fibrosis and cancer, where it may contribute to invasiveness and metastatic behavior of tumor cells^25^. The importance of EMT in normal and disease conditions has raised much interest in the underpinning and regulation of EMT. The mechanisms that regulate EMT remain to be fully investigated.

This study reveals novel isoform-specific functions for TCF7L2 in EMT and organoid morphogenesis regulation. Using three-dimensional epithelial cell-derived organoid models, gain and loss of function studies reveal that whereas E-isoforms suppress, the M or S isoforms promote EMT. We also find that the β-catenin domain within TCF7L2 is not required for the antagonistic functions of the TCF7L2 isoforms, suggesting that Wnt-β-catenin signaling may not regulate TCF7L2 role in EMT. Importantly, we find that the secreted factor transforming growth factor beta (TGFβ), a potent inducer of EMT, reduces the protein abundance of TCF7L2 isoforms protein ratio of E to S/ M in order to promote EMT in epithelial cells-derived organoids. Collectively, our study points to an isoform-specific functions for TCF7L2 mediated by TGFβ signaling in regulating EMT-like effects in epithelial cell-derived organoids.

## Results

### Expression of TCF7L2 isoforms in mammary epithelial cells

Exons 13–16 within TCF7L2 pre-mRNA can be alternatively spliced giving rise to several iterations of C-terminal variant E, M, and S isoforms (Fig. S1A). Exon 4 inclusion or exclusion contributes to additional variations in these TCF7L2 isoforms. A key question that has remained to be investigated is the functional significance of the TCF7L2 E, M, and S isoforms. We sought to determine TCF7L2 role in EMT, which is fundamental for shaping the developing organism and contributes to homeostasis and diseases. We employed the mouse mammary epithelial NMuMG cells, a widely used cellular model to study EMT including in the context of three-dimensional (3D) culture system^26–29^. First, we characterized TCF7L2 isoforms profile in lysates of subconfluent NMuMG cell monolayers. Immunoblotting analyses revealed anti-TCF7L2-immunoreactive-protein species at 75 and 60 kD, potentially corresponding to the E and S/M isoforms, respectively, with the first showing a higher abundance than the latter (Fig. S1A and S1B). RT-PCR analyses indicated that the C-variant E2, E3, E4, M1, M2, and S2 isoforms of TCF7L2, with or without exon 4, may be expressed in NMuMG cells (Fig. S1E and S1F).

### TCF7L2 regulates EMT-like responses in organoids in an isoform-specific manner

**Fig. 1:**
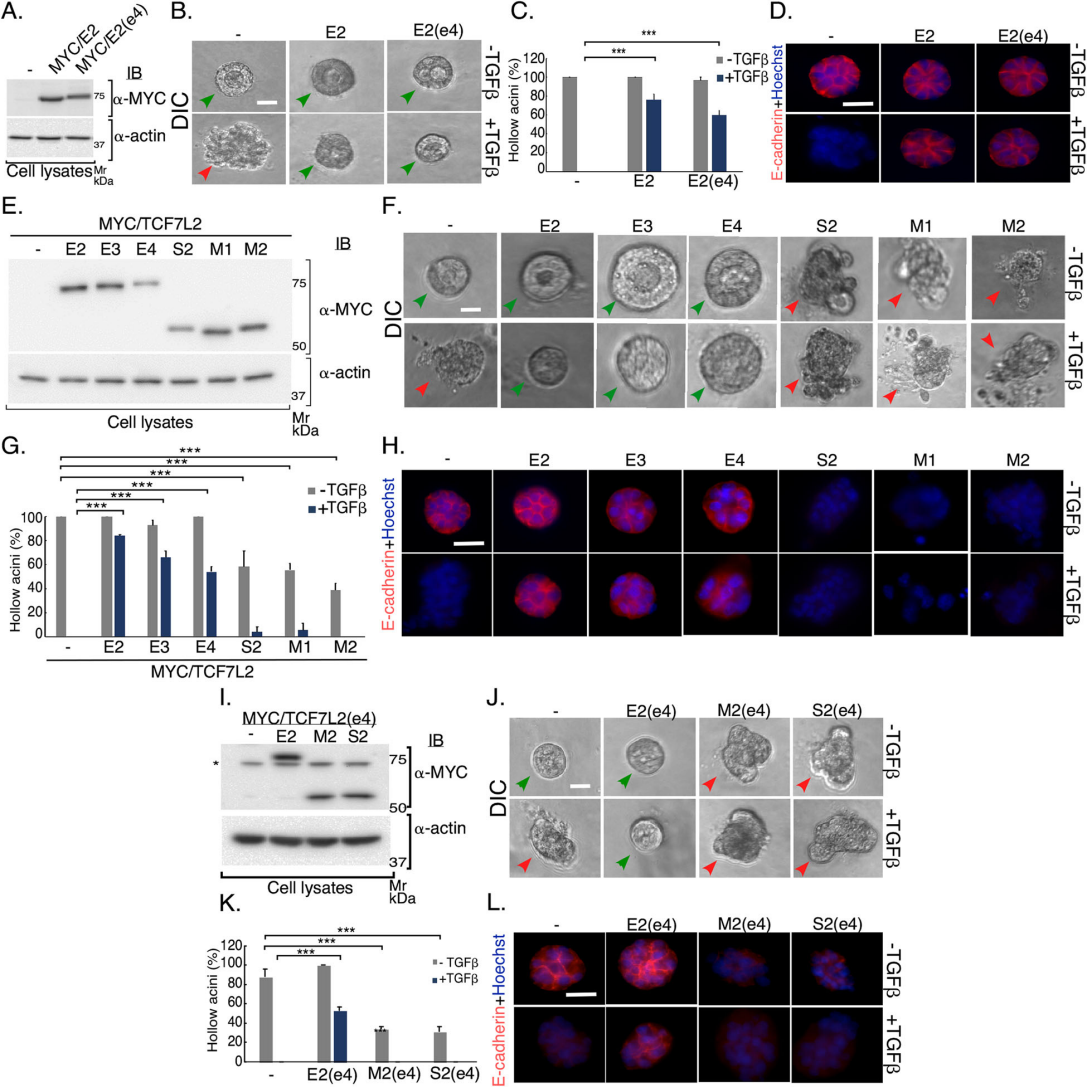
TCF7L2 isoforms differentially regulate the morphogenesis of three-dimensional mammary epithelial cell-derived organoids. **a**–**d** *T*CF7L2E2 expression suppresses TGFβ-induced EMT of 3D-NMuMG cell-derived organoids. **a** Lysates of NMuMG cells transfected with the vector control or a plasmid-containing cDNA encoding MYC-tagged TCF7L2E2 protein with (E2-e4) or without (E2) exon 4-encoded amino acids, were subjected to immunoblotting with MYC or actin antibody, with the latter used as loading control. **b** Representative DIC images of 8-day-old untreated or TGFβ-stimulated NMuMG cells transfected as described in 1 A. Green and red arrowheads indicate hollow center and filled/deformed acini, respectively. Scale bar indicates 50 μm. **c** Bar graph of average percent (±SEM) of hollow acinar of untreated or TGFβ-stimulated 8-day old 3D-organoids derived from NMuMG cells transfected as in 1 A from three biological replicates of the experiment, including the one shown in 1B (ANOVA: ***P ≤ 0.001). Expression of TCF7L2E2, with or without exon 4, suppressed the ability of TGFβ to promote acini filling of epithelial cell-derived organoids. **d** Representative fluorescence microscopy scans of E-cadherin- (E-cadherin antibody-red) and nuclear- (Hoechst 33258, blue) stained formalin-fixed untreated or TGFβ-stimulated 8-day-old 3D-organoids derived from mammary epithelial cells transfected and subjected to three-dimensional culture as described in 1**a**–**c**. Scale bar indicates 50 μm. TCF7L2E2 expression suppressed TGFβ‘s ability to decrease the protein abundance and/or mislocalization of E-cadherin, considered as key hallmarks of TGFβ-induced EMT. **e**–**h** E vs M/S TCF7L2 isoform expression has opposing effects on EMT induction of NMuMG cell-derived organoids. **e** Lysates of NMuMG cells transfected with vector control or plasmid-containing cDNA encoding MYC-tagged E2, E3, E4, S2, M1, or M2 TCF7L2 isoform, were subjected to MYC or actin immunoblotting, with the latter as a loading control. **f** Representative DIC images of untreated or TGFβ-stimulated 8-day old 3D-organoids derived from NMuMG cells transfected as described in 1E. Green and red arrowheads indicate hollow center and filled/deformed acini, respectively. Scale bar indicates 50 μm. **g** Bar graph of average percent (±SEM) of hollow acinar of untreated or TGFβ-stimulated 8-day old 3D-organoids derived from NMuMG cells transfected as in 1E from three biological replicates of the experiment, including the one shown in 1 F (ANOVA: ***P ≤ 0.001). Like E2, E3, or E4 expression suppressed TGFβ-induced acini filling, whereas S2, M1, or M2 expression promoted acini filling even in the absence of exogenous TGFβ. **h** Representative fluorescence microscopy scans of E-cadherin- (E-cadherin antibody, red) and nuclear- (Hoechst 33258, blue) stained formalin-fixed untreated or TGFβ-stimulated 8-day-old 3D-organoids derived from NMuMG cells transfected and subjected to three-dimensional culture as described in 1**e** and **f**. Scale bar indicates 50 μm. Similar to E2, E3, and E4 expression suppressed TGFβ-mediated loss and mislocalization of E-cadherin. M1, M2, or S2 expression induced loss and mislocalization of E-cadherin even in absence of exogenous TGFβ. **i**–**l** Expression of TCF7L2M or TCF7L2S isoforms, with or without exon 4, regulates EMT. **i** Lysates of NMuMG cells transfected with vector control, or plasmid-containing cDNA encoding MYC-tagged E2, M2, or S2 TCF7L2 isoforms with exon 4-encoded amino acids (E2-e4, M2-e4, and S2-e4) were subjected to MYC or actin immunoblotting, with the latter as a loading control. Asterix (*) indicates a non-specific immunoreactive band. **j** Representative DIC images of untreated or TGFβ-stimulated 8-day-old 3D-organoids derived from NMuMG cells transfected as in 1I. Green and red arrowheads indicate hollow center and filled/deformed acini, respectively. Scale bar indicates 50 μm. **k** Bar graph of average percent (±SEM) of hollow acinar untreated or TGFβ-stimulated 8-day old 3D-organoids derived from NMuMG cells transfected as described in 1I from three biological replicates of the experiment, including the one shown in 1 J (ANOVA: ***P ≤ 0.001). **l** Representative fluorescence microscopy scans of E-cadherin- (E-cadherin antibody, red) and nuclear- (Hoechst 33258, blue) stained formalin-fixed untreated or TGFβ-treated 8-day old 3D-organoids derived from NMuMG cells transfected and subjected to three-dimensional culture as described in 1**i**–**k**. Scale bar indicates 50 μm. E2 and M2/S2, with or without exon 4, prevent and promote, respectively, alterations in E-cadherin abundance/localization.

That the E, S, and M TCF7L2 isoforms are expressed in the mammary epithelial cells raised the key question of their functional significance in these cells. We, first, subcloned and sequence-verified TCF7L2 E2, E3, E4, M1, M2, and S2 cDNA into an expression plasmid (M&M, and Fig. S1C–S1F). We, then, used a 3D-culture model, employing Matrigel as a source of extracellular matrix (ECM), to determine the role of TCF7L2 isoforms in EMT-like responses^27,29^. 3D culturing of cells supports the organization of proliferating epithelial cells into multicellular structures characterized by hollow spherical or acinar morphology with apical/basal polarity, akin to glandular epithelial tissues in vivo, and that are also termed ‘acini’ or ‘organoids’^26,27,30^. EMT induction by the secreted protein TGFβ, a potent inducer of EMT in development and disease including cancer^21,23,25^, can lead to many cellular changes including cell–cell and cell–matrix detachments, apical–basal polarity loss, and invasion, manifesting as hollow-lumen filling and deformation, including invasiveness and budding, of the organoids. The epithelial marker and adherens junctional protein E-cadherin contributes to epithelial tissue polarity and morphology^20^. Loss and/or mislocalization of E-cadherin is considered a key hallmark of EMT (Fig. 1d)^31^.

We found that expression of TCF7L2E2 isoform, with or without exon 4, suppressed TGFβ-induced filling and deformation of 3D-NMuMG cell-derived acini (Figs. 1a–c and S2A). In addition, immunocytochemical analysis showed that overexpression of the TCF7L2E2 isoform blocked TGFβ-induced reduction in the protein abundance and/or mislocalization of E-cadherin in NMuMG cell-derived acini (Fig. 1d). Together, these data suggested that TCF7L2E2 may negatively regulate the induction of EMT-like effects in epithelial cell-derived organoids. These data also suggested that overall, exon 4-encoding region may not affect E-isoforms suppression of EMT.

Next, we compared the effect of expression of TCF7L2 E3, E4, M1, M2, or S2 to that of the E2 isoform on acinar morphology in the absence or presence of TGFβ. We found, similar to TCF7L2E2, overexpressed E3 and E4 isoforms suppressed TGFβ-induced EMT (Figs. 1e–g and S2B). In contrast, overexpressed TCF7L2 M1, M2, and S2 led to filling and deformation (Figs. 1e–g and S2B), and reduced protein level and mislocalization of E-cadherin (Fig. 1h), in the NMuMG cell-derived organoids, even in the absence of TGFβ, suggesting that these isoforms might promote EMT-like effects. Our data, also, suggested that the EMT-like effects induced by the TCF7L2 M2 and S2 isoforms may not be affected by exon 4-coding peptide (Figs. 1i–l and S2C). Collectively, these data suggest that C-terminal TCF7L2 E variants may suppress, while the M and S variants may induce EMT-like effects in epithelial cells.

### TCF7L2 C-variant isoforms may regulate EMT independently of Wnt-β-catenin signaling

**Fig. 2:**
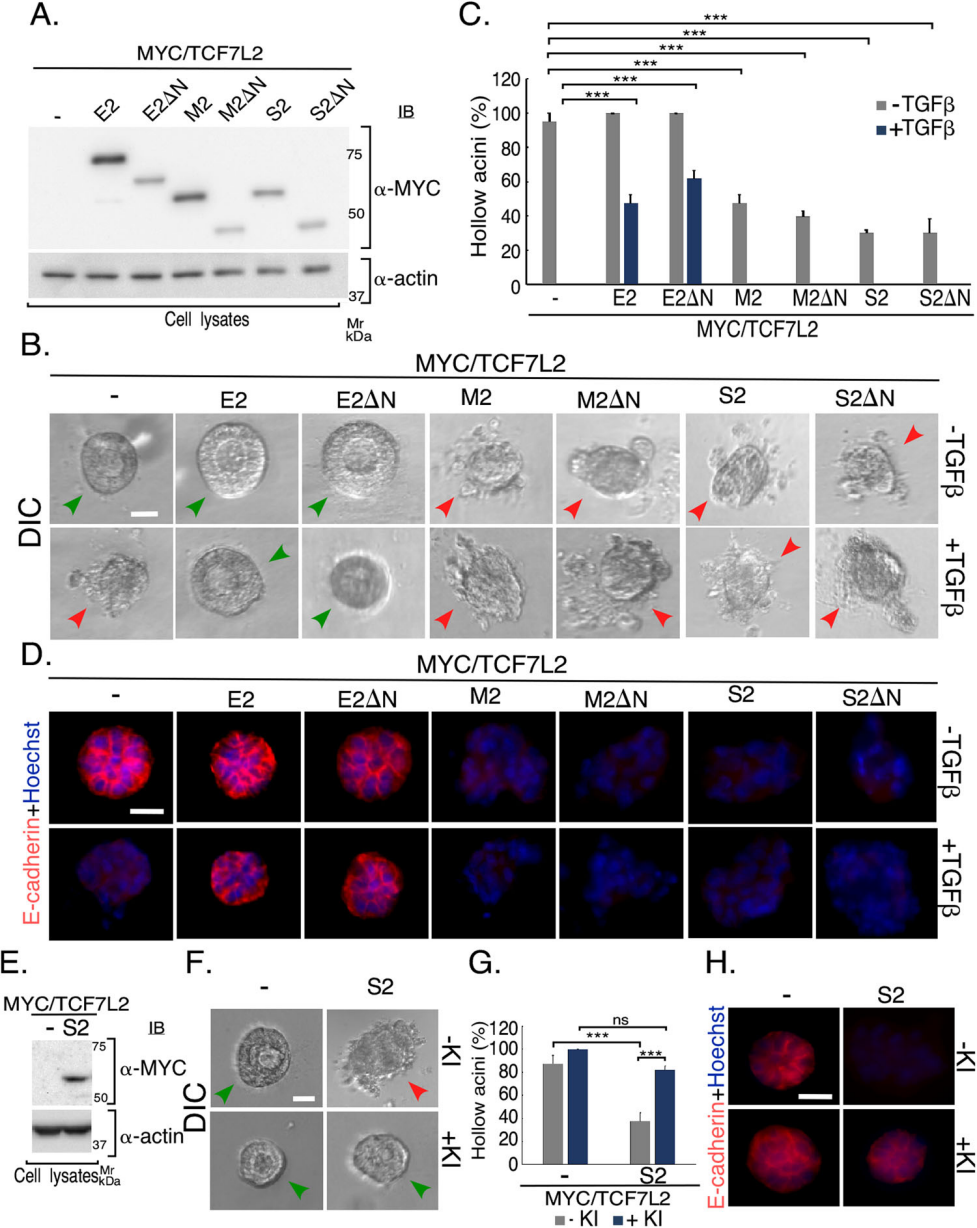
TCF7L2 isoforms may act in a β-catenin-independent manner to regulate TGFβ-induced EMT in 3D-mammary epithelial cell-derived organoids. **a**–**d** Deletion of β-catenin binding domain does not impact the ability of expressed TCF7L2 isoforms to regulate EMT. **a** Lysates of NMuMG cells transfected with vector control or a plasmid-containing cDNA encoding MYC-tagged full length or a β-catenin binding domain deletion mutant (ΔN) version of TCF7L2 E2, M2, or S2 were subjected to MYC and actin immunoblotting, with the latter as a loading control. **b** Representative DIC images of untreated or TGFβ-stimulated 8-day-old 3D-organoids derived from NMuMG cells transfected as described in 2**a**. Green and red arrowheads indicate hollow center and filled/deformed acini, respectively. Scale bar indicates 50 μm. **c** Bar graph of average percent (±SEM) of hollow acinar untreated or TGFβ-stimulated 8-day-old 3D-organoids derived from NMuMG cells transfected as in 2**a** from three biological replicates of the experiment, including the one shown in 2**b** (ANOVA: ***P ≤ 0.001). **d** Representative fluorescence microscopy scans of E-cadherin- (E-cadherin antibody, red) and nuclear- (Hoechst 33258, blue) stained formalin-fixed untreated or TGFβ-stimulated 8-day-old 3D-organoids derived from NMuMG cells transfected and subjected to three-dimensional culture as described in 2A-2C. Scale bar indicates 50 μm. **e**–**h** S2 expression promotes EMT in a TGFβ signaling-dependent manner. **e** Lysates of NMuMG cells transfected with vector control or a plasmid-containing cDNA encoding MYC/TCF7L2S2 were subjected to MYC and actin immunoblotting, with the latter as a loading control. **f** Representative DIC images of 8-day-old 3D-organoids derived from NMuMG cells transfected as described in 2**e**. The organoid cultures were incubated with the TGFβ signaling inhibitor SB431542 (+KI), a small molecule inhibitor of the ser/thr kinase activity of the TGFβ type I receptor, or with the vehicle (−KI). Green and red arrowheads indicate hollow center and filled/deformed acini, respectively. Scale bar indicates 50 μm. **g** Bar graph of average percent (±SEM) of hollow acinar vehicle-treated (−KI) or TGFβ signaling inhibitor-treated (+KI) 8-day-old 3D-organoids derived from mammary epithelial cells transfected as described in 2**e** from three biological replicates of the experiment, including the one shown in 2**f** (ANOVA: ***P ≤ 0.001, ns not significant). Inhibition of basal TGFβ signaling reversed the ability of TCF7L2S2 isoform to promote acini filling. **h** Representative fluorescence microscopy scans of E-cadherin (E-cadherin antibody, red) and nuclear (Hoechst 33258, blue) stained formalin-fixed 8-day old 3D-organoids derived from NMuMG cells transfected and treated as described in 2**e**–**g**. Scale bar indicates 50 μm. Inhibition of basal TGFβ signaling reversed the ability of TCF7L2S2 isoform to promote loss and mislocalization of E-cadherin.

TCF7L2 contributes to the canonical Wnt-β-catenin-signaling pathway and responses, raising the question whether Wnt signaling influences the isoform-specific effects of TCF7L2 in EMT. TCF7L2 associates via its β-catenin binding domain with the transcriptional regulator β-catenin to regulate Wnt-dependent gene expression and responses^3,32^. We, thus, determined the effect of deletion of the β-catenin binding domain on the ability of TCF7L2 isoforms to regulate EMT in NMuMG cell-derived acini. Deletion of the β-catenin domain did not affect the ability of the TCF7L2E2 isoform to inhibit TGFβ-induced EMT in 3D-epithelial cell-derived acini (Figs. 2a–d and S3A). We, also, found the β-catenin binding domain deletion did not seem to alter S2 or M2 TCF7L2 isoform promotion of EMT-like responses (Figs. 2a–d and S3A). Thus, these data suggest that the canonical Wnt-β-catenin signaling may not be essential for TCF7L2 isoform-specific functions in EMT-like effects. On the other hand, we found blocking endogenous TGFβ signaling in the NMuMG cell-derived organoids suppressed the ability of the M and S isoforms to promote filling and morphological deformation, and loss or mislocalization of E-cadherin in these acini (Figs. 2e–h and S3B and data not shown). Together, these data suggest that the opposing effects on EMT of overexpressed C-terminal isoform variants of TCF7L2 in 3D-epithelial cell-derived organoids may not require β-catenin-mediated signaling.

### Endogenous TCF7L2 acts in an isoform-specific manner to regulate acini morphology

**Fig. 3:**
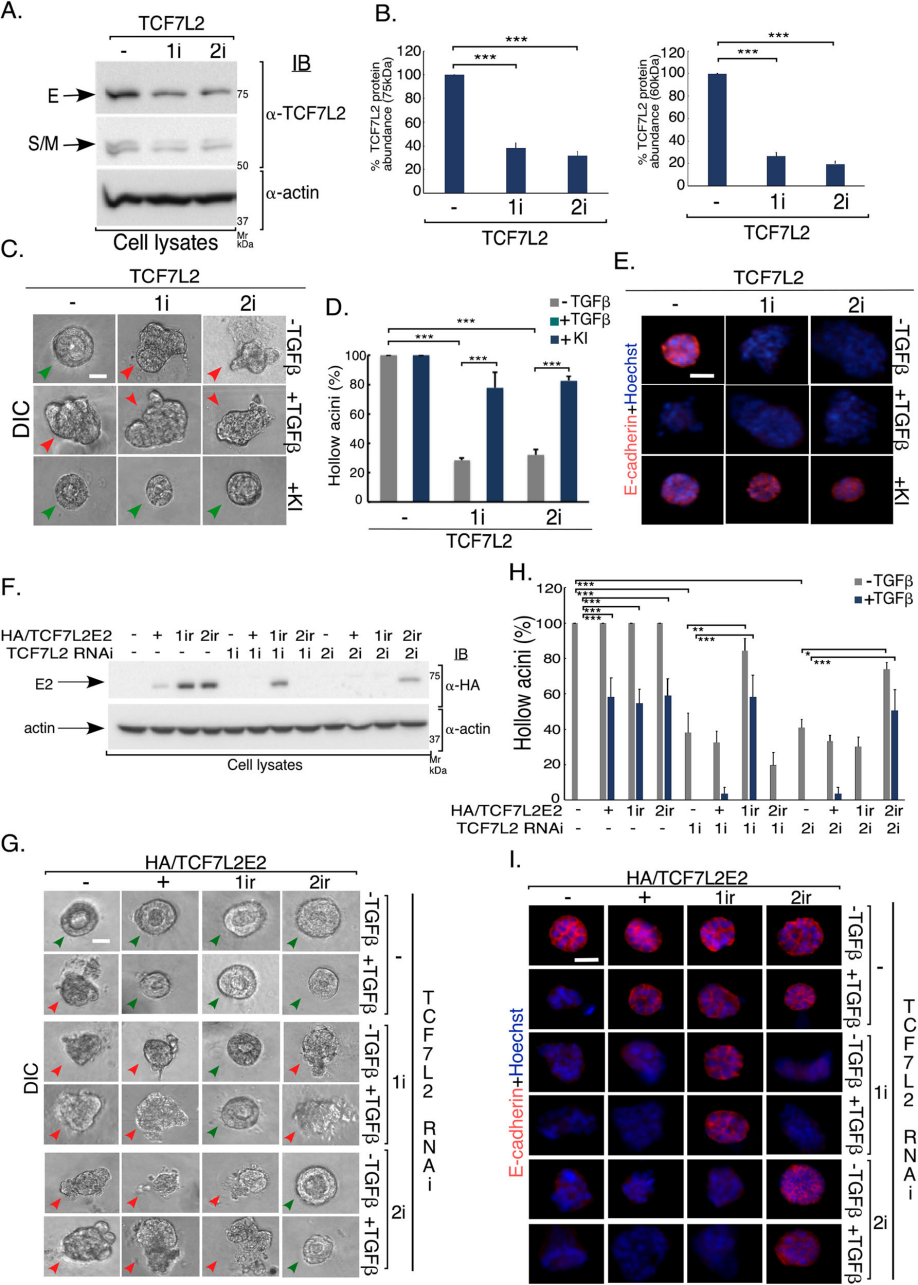
Endogenous TCF7L2 acts in an isoform-dependent manner to regulate mammary epithelial-acini morphology. **a** Lysates of NMuMG cells transfected with an RNAi vector control (−) or an RNAi plasmid encoding shRNA1 (TCF7L2-1i) or shRNA2 (TCF7L2-2i) targeting two distinct regions of TCF7L2 mRNA (Table 2), were subjected to TCF7L2 or actin immunoblotting, the latter as a loading control. **b** Bar graph depicts average of actin-normalized protein abundance of 75 kDa (E isoforms) and 60 kDa (M and S isoforms) (±SEM) of TCF7L2-immunoreactive protein species in lysates of NMuMG cells transfected as in 3**a**, and expressed relative to the RNAi vector control from four biological replicates of the experiment, including the one shown in 3**a** (ANOVA: ***P ≤ 0.001). shRNA1 and shRNA2 reduced the protein abundance of endogenous TCF7L2. **c** Representative DIC images of 8-day-old untreated, KI-treated, or TGFβ-treated 3D-organoids derived from NMuMG cells transfected and analyzed as described in 3**a** and **b**. Green and red arrowheads indicate hollow center and filled/deformed acini, respectively. Scale bar indicates 50 μm. **d** Bar graph of average percent (±SEM) of hollow acinar untreated, KI, or TGFβ-incubated 8-day-old 3D-organoids derived from NMuMG cells transfected as described in 3**a** from three biological replicates of the experiment, including the one shown in 3**c** (ANOVA: ***P ≤ 0.001). TCF7L2-1i and 2i promoted acini filling even in the absence of TGFβ, and which was reversed by TGFβ signaling inhibition (KI). **e** Representative fluorescence microscopy scans of E-cadherin- (E-cadherin antibody, red) and nuclear- (Hoechst 33258, blue) stained formalin-fixed 8-day-old untreated, KI or TGFβ-treated 3D-organoids derived from NMuMG cells transfected and subjected to three-dimensional culture as described in 3**a**–**d**. Scale bar indicates 50 μm. TCF7L2-1i and 2i promoted loss and mislocalization of E-cadherin even in the absence of TGFβ, which were reversed by TGFβ signaling inhibition (KI). **f** Lysates of NMuMG cells transfected with RNAi vector control (−), or one expressing TCF7L2-1i or 2i, together with a control expression vector, or a plasmid-containing cDNA encoding hemagglutinin (HA)-tagged TCF7L2E2 version that is sensitive to both TCF7L2-1i and −2i (+), only to TCF7L2-2i (1ir), or only TCF7L2-1i (2ir) were subjected to HA and actin immunoblotting, with the latter as a loading control. **g** Representative DIC images of 8-day-old untreated or TGFβ-stimulated 3D-organoids derived from NMuMG cells transfected as described in 3**f**. Green and red arrowheads indicate hollow center and filled/disrupted acini, respectively. Scale bar indicates 50 μm. **h** Bar graph of average percent (±SEM) of hollow acinar untreated or TGFβ-stimulated 8-day-old 3D-organoids derived from NMuMG cells transfected as described in 3**f** from four biological replicates of the experiment, including the one shown in 3**g** (ANOVA: *P ≤ 0.05, **P ≤ 0.01, ***P ≤ 0.001). **i** Representative fluorescence microscopy scans of E-cadherin (E-cadherin antibody, red) and nuclear (Hoechst 33258, blue) stained formalin-fixed 8-day-old untreated or TGFβ-incubated 3D-organoids derived from NMuMG cells transfected and subjected to three-dimensional culture as described and shown in 3**f** and 3**g**. Scale bar indicates 50 μm. TCF7L2 E2-1ir and E2-2ir inhibited, respectively, the ability of TCF7L2 -1i and -2i to promote acini filling, deformation and loss/mislocalization of E-cadherin in the absence and presence of TGFβ.

We next used RNA interference (RNAi) to characterize the role of endogenous TCF7L2 in EMT-like effects in epithelial cell-derived acini. We generated two short hairpin RNA (shRNA)-expressing constructs RNA1i and RNA2i that target two distinct constitutive regions of TCF7L2 mRNA (Table 2 and Fig. S1A), to knockdown all TCF7L2 isoforms including E, M, and S isoforms in NMuMG cells (Fig. 3a, b). Knockdown of TCF7L2 promoted EMT-like behavior in the NMuMG cell-derived acini even in the absence of TGFβ as compared to the vector control-transfected cells (Figs. 3c–e and S4A). The TCF7L2 knockdown-induced EMT-like responses were reversed by suppression of endogenous TGFβ signaling. Importantly, expression of RNAi-resistant protein TCF7L2E2-1ir or TCF7L2E2-2ir reversed EMT induction by the respective TCF7L2 shRNA, excluding the possibility that the RNAi-induced EMT phenotype was due to off target effects (Figs. 3f–i and S4B). On the other hand, expression of the corresponding TCF7L2M2-1ir or TCF7LS2-1ir rescue proteins did not reverse the EMT responses induced by TCF7L2 shRNA1 (Fig. S4C–S4G). Altogether, these data are consistent with the idea that at endogenous levels, TCF7L2E isoforms suppress EMT.

**Fig. 4:**
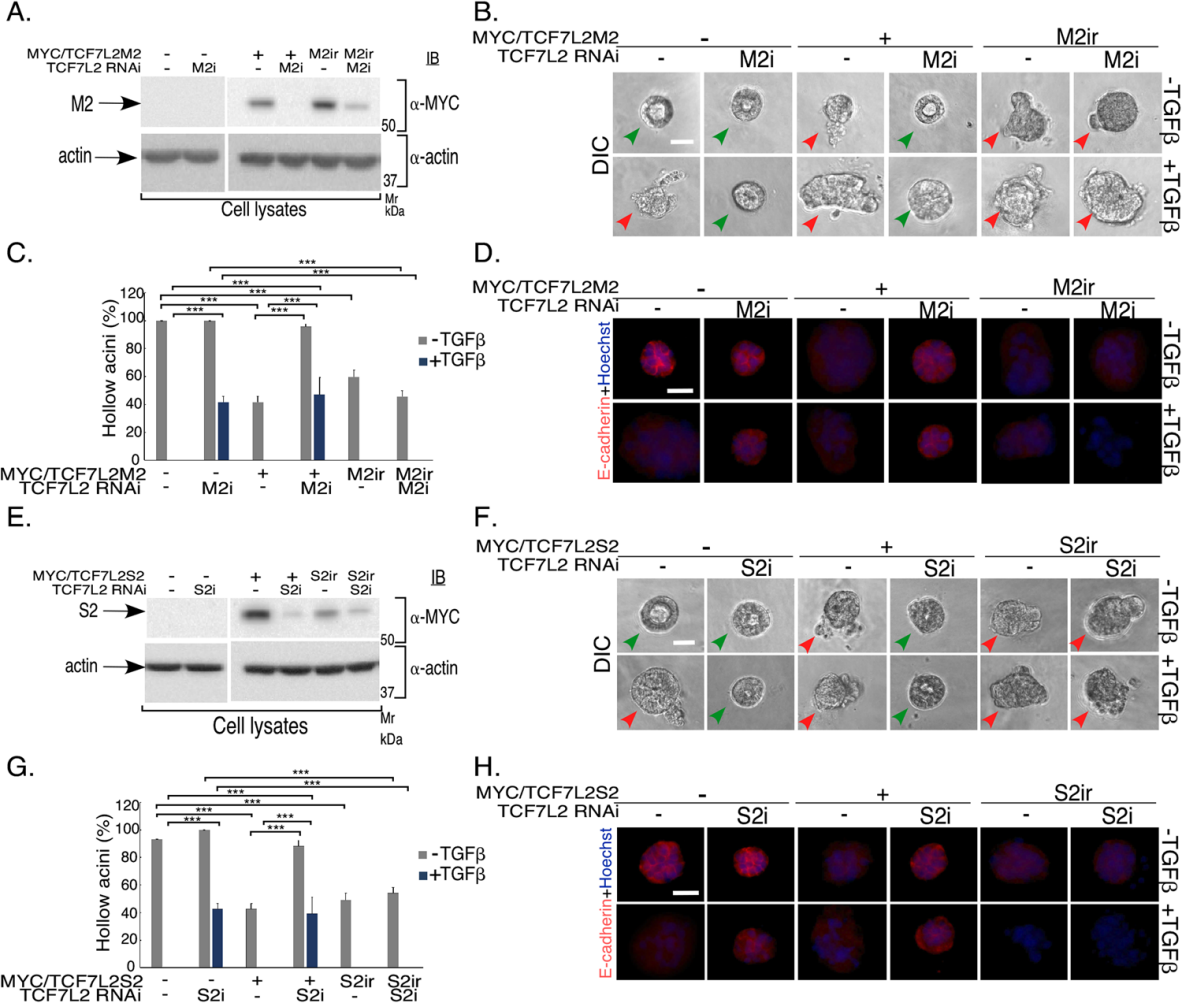
Endogenous M and S isoforms of TCF7L2 promote EMT in 3D-mammary epithelial cell-derived organoids. **a**–**d** Knockdown of endogenous TCF7L2M2 suppresses TGFβ-induced EMT. **a** Lysates of NMuMG cells transfected with an RNAi vector control plasmid (−), or a TCF7LM2i plasmid expressing shRNA against TCF7L2M2 (M2i), together with control vector (−) or a plasmid expressing a MYC/TCF7L2M2 protein that is sensitive (+) or resistant (M2ir) to TCF7L2M2i, were subjected to MYC or actin immunoblotting, with the latter used as loading control. **b** Representative DIC images of 8-day old untreated or TGFβ-stimulated 3D-organoids derived from NMuMG cells transfected as described in 4**a**. Green and red arrowheads indicate hollow center and filled/deformed acini, respectively. Scale bar indicates 50 μm. **c** Bar graph of average percent (±SEM) of hollow acinar untreated or TGFβ-stimulated 8-day-old 3D-organoids derived from NMuMG cells transfected as described in 4**a** from three biological replicates of the experiment, including the one shown in 4**b** (ANOVA: ***P ≤ 0.001). TCF7L2M2i reduced TGFβ-induced acini filling, which was reversed by expression of the RNAi rescue protein TCF7L2M2-M2ir. **d** Representative fluorescence microscopy scans of E-cadherin- (E-cadherin antibody, red) and nuclear- (Hoechst 33258, blue) stained formalin-fixed 8-day-old untreated or TGFβ-stimulated of 3D-organoids derived from NMuMG cells transfected and subjected to three-dimensional culture as described and shown in 4**a**–**b**. Scale bar indicates 50 μm. Knockdown of endogenous TCF7L2M2 suppressed reduction and mislocalization of E-cadherin by TGFβ, which was reversed by expression of the RNAi rescue protein TCF7L2M2-M2ir. **e**–**h** Knockdown of endogenous TCF7L2S2 suppresses TGFβ-induced EMT. **e** Lysates of NMuMG cells transfected with an RNAi vector control plasmid (−), or a TCF7LS2i plasmid expressing shRNA against TCF7L2S2 (S2i), together with a control vector (−) or a plasmid expressing a MYC/TCF7L2S2 protein that is sensitive (+) or resistant (S2ir) to TCF7L2S2i, were subjected to MYC or actin immunoblotting, with the latter used as loading control. **f** Representative DIC images of 8-day old untreated or TGFβ-stimulated 3D-organoids derived from NMuMG cells transfected as described in 4**e**. Green and red arrowheads indicate hollow center and filled/deformed acini, respectively. Scale bar indicates 50 μm. **g** Bar graph of average percent (±SEM) of hollow acinar untreated or TGFβ-stimulated 8-day old 3D-organoids derived from NMuMG cells transfected as described in 4**e** from three biological replicates of the experiment, including the one shown in 4**f** (ANOVA: ***P ≤ 0.001). TCF7L2S2i reduced TGFβ-induced acini filling, which was reversed expression of the RNAi rescue protein TCF7L2S2-S2ir. **h** Representative fluorescence microscopy scans of E-cadherin- (E-cadherin antibody, red) and nuclear- (Hoechst 33258, blue) stained formalin-fixed of untreated or TGFβ-stimulated 8-day-old 3D-organoids derived from NMuMG cells transfected and subjected to three-dimensional culture as described and shown in 4**e**–**f**. Scale bar indicates 50 μm. Knockdown of endogenous TCF7L2S2 suppressed reduction and mislocalization of E-cadherin by TGFβ, which was reversed by expression of the RNAi rescue protein TCF7L2S2-S2ir.

We next generated the RNAi plasmids RNA-M2i and RNA-S2i encoding TCF7L2 shRNA-M2i and shRNA-S2i to selectively knockdown the TCF7L2 M2 and S2 isoforms, respectively (Table 2, Figs 4a, e, S1A). Remarkably, TCF7L2M2 knockdown suppressed acini TGFβ-induced filling and deformation, and E-cadherin loss or mislocalization (Figs. 4b–d and S5A). Similarly, TCF7L2S2 knockdown reversed TGFβ-induced EMT-like effects (Figs. 4f–h, and S5B). Further, expression of RNAi-resistant protein TCF7L2M2-M2ir and TCF7L2S2-S2ir reversed EMT suppression by shRNA-M2i and shRNA-S2i, respectively, suggesting that endogenous TCF7L2 M and S isoforms promote TGFβ-induced EMT in NMuMG cell-derived organoids (Figs. 4b–d, f–h and S5A, S5B). Taken together, our data demonstrate that endogenous TCF7L2 regulates EMT-like effects in an isoform-specific manner. Whereas TCF7L2M/S isoforms promote, TCF7L2E isoforms suppress EMT-like effects in epithelial cells.

### TGFβ regulates TCF7L2 abundance in epithelial cells undergoing EMT

**Fig. 5:**
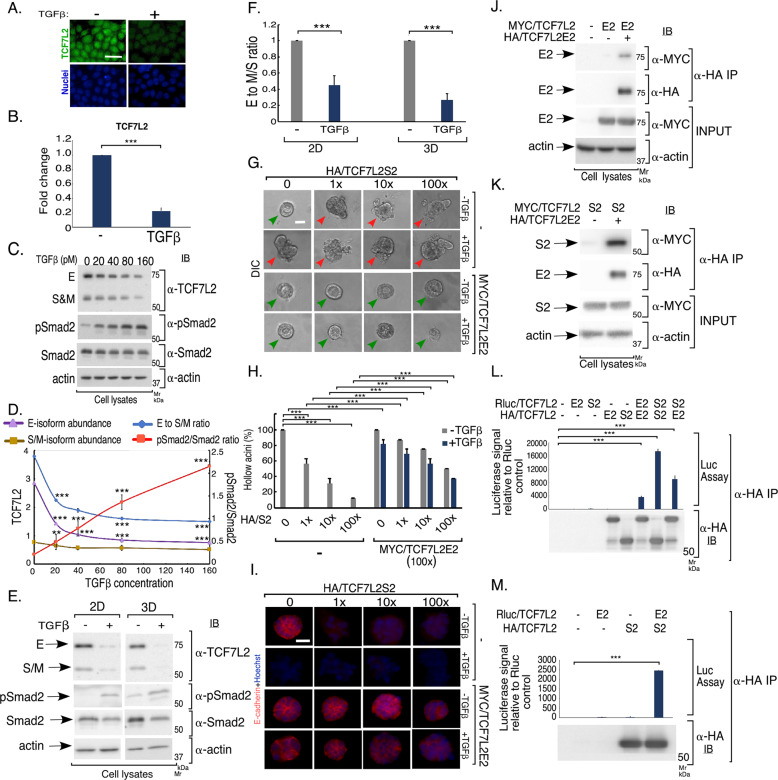
TGFβ downregulates the protein ratio of TCF7L2 E to M/S in mammary epithelial cells. **a** Representative fluorescence microscopy scans of TCF7L2- (TCF7L2 antibody, green) and nuclear-(Hoechst 33258, blue) stained formalin-fixed subconfluent monolayers of NMuMG cells left untreated or incubated with 100 pM TGFβ for 48 h. The images are from one of two biological replicates of the experiment. TCF7L2 localizes mainly in the nucleus. TCF7L2 signal intensity was quantified using Image J software analyses. TGFβ led to an average of approximately 75% reduction in the protein abundance of TCF7L2 in NMuMG cells undergoing EMT.**b** TGFβ signaling downregulates TCF7L2 transcript level. **b** Bar graph of average (±SEM) of relative GAPDH-normalized TCF7L2 mRNA determined by quantitative real time PCR (qRT-PCR) of cDNA derived from untreated or TGFβ-stimulated NMuMG cells from four biological replicates of the experiment (ANOVA: ***P ≤ 0.001). **c**, **d** TGFβ reduces the protein proportion of E to S/M in NMuMG cells. **c** TCF7L2, pSmad2, Smad2/3 and actin immunoblotting of lysates of NMuMG cells incubated with increasing concentrations of TGFβ for 48 h. **d** XY Plot of mean (±SEM) of the protein abundance of E isoforms (violate-triangle and line), S/M-isoforms (golden-square and line), and protein ratio of E to S/M (blue-circle and line) (left *Y*-axis) and pSmad2 expressed relative to Smad2 (red-square and line) (right *Y*-axis) versus TGFβ concentration (*X*-axis) from six biological replicates of the experiment, including the one shown in 5**c** (ANOVA: **P ≤ 0.01, ***P ≤ 0.001, as compared to respective untreated control). **e**, **f** TCF7L2 protein isoforms profiling in NMuMG-derived organoids and effect of TGFβ stimulation. **e** TCF7L2, pSmad2, Smad2/3 and actin immunoblotting of lysates of 48h-monolayer (2D) or 8-day organoid (3D) cultures of NMuMG cells left untreated or stimulated with TGFβ. **f** Bar graph of average (±SEM) of the protein ratio of E isoforms to that of the M/S isoforms of TCF7L2 from four biological replicates of the experiment, including the one shown in 5**e** (ANOVA: ***P ≤ 0.001, as compared to respective untreated control). **g**–**i** TCF7L2E2 and TCF7L2S2 coregulate morphogenesis of 3D-mammary epithelial cell-derived organoids. **g** Representative DIC images of untreated or TGFβ-stimulated 8-day-old 3D-organoids derived from NMuMG cells transfected as described in Fig. S6D. Green and red arrowheads indicate hollow center and filled/deformed acini, respectively. Scale bar indicates 50 μm. **h** Bar graph of average percent (±SEM) of hollow acinar untreated or TGFβ-stimulated 8-day-old 3D-organoids derived from NMuMG cells transfected as in Fig. S6D from three biological replicates of the experiment, including the one shown in 5**g** (ANOVA: ***P ≤ 0.001). **i** Representative fluorescence microscopy scans of E-cadherin- (E-cadherin antibody, red) and nuclear- (Hoechst 33258, blue) stained formalin-fixed untreated or TGFβ-stimulated 8-day-old 3D-organoids derived from NMuMG cells transfected and subjected to three-dimensional culture as described in Figs. S6D and 5G. Scale bar indicates 50 μm. **j**–**m** TCF7L2 isoforms form homo and hetero-oligomers in cells. Lysates of 293 T cells transfected with vector control or one expressing MYC/TCF7L2 E2 (J) or S2 (K) without (−) or with HA/TCF7L2E2 (+) (J and K) were subjected to anti-HA immunoprecipitation (IP), followed by sequential MYC and HA immunoblotting. Lysates were also subjected to MYC, HA and actin immunoblotting, with the latter used as loading control. TCF7L2E2 formed E2-homo-oligomers (**j**) and S2-hetero-oligomers (**k**). Representative scans are shown in each **j** and **k** from one of two biological replicates of the experiment. Lysates of 293 T cells transfected with a plasmid-containing cDNA encoding the Renilla luciferase protein (Rluc) alone (−) (**l** and **m**) or in fusion with TCF7L2E2 (E2) (**l** and **m**) or TCF7L2S2 (S2) (**l**), together with a vector control (−) or a plasmid expressing HA/TCF7L2E2 (**l**) or HA/TCF7L2S2 (**l** and **m**), were anti-HA immunoprecipitated (α-HA-IP) followed by subjecting 90% of the immunocomplexes to Renilla luciferase activity measurement (Luc assay) and the rest to anti-HA immunoblotting. 10% of each lysate was subjected to luciferase measurement as a relative indicator of protein abundance of the luciferase gene product or input. Upper panels- bar graphs showing average (±SEM) input-normalized luciferase values in the HA-immunocomplexes from three (**l**) and five (**m**) biological replicates of the experiment (ANOVA: ***P ≤ 0.001). Lower panels- HA immunoblot of the HA immunoprecipitation from a representative biological replicate of the experiments shown in **l** and **m**.

The identification of a novel isoform-specific function of TCF7L2 in EMT led us next to the fundamental question of how TCF7L2 might be regulated in EMT. We first determined if EMT affects the abundance and subcellular localization of TCF7L2 in epithelial cells. Immunocytochemistry analyses revealed that TCF7L2 is predominately nuclear in NMuMG epithelial cells (Fig. 5a). Remarkably, the protein abundance of TCF7L2 was reduced by ~75% in cells undergoing TGFβ-induced EMT (Fig. 5a). qRT-PCR analyses revealed that the abundance of TCF7L2 mRNA was reduced by close to 80% in cells undergoing EMT (Figs. 5b and S6A, S6B). Interestingly, TGFβ signaling activation decreased the protein abundance of TCF7L2 E isoforms to a greater extent than that of the S/M isoforms in cells leading to a decrease in the protein proportion of TCF7L2 E isoforms to that of the S/M isoforms (Fig. 5c, d). In other experiments, we found that the TCF7L2 E isoforms are also expressed at a higher relative abundance compared to the S/M isoforms in the NMuMG cell-derived organoids (Fig. 5e, f). Importantly, increased TGFβ signaling (Fig. 5e, second and third panels, respectively), reduced the protein abundance of the TCF7L2 isoforms with an overall E to M/S isoform reduction in protein ratio in the NMuMG-derived organoids (Fig. 5e, f). We, also, found that TGFβ signaling increased the protein abundance of the EMT-inducing transcription factor Smad interacting protein (SIP1), or Zeb2, while decreasing the SIP1-repressed target E-cadherin suggesting TGFβ-induced EMT-like effects in these organoids (Fig. S6C). In studies investigating the E-S/M functional interplay, we found expression of the TCF7L2E2 suppressed TCF7L2S2 promotion of EMT (Figs. 5g–i and S6D, S6E). Reciprocal coimmunoprecipitations (Co-IP) revealed that E2 and S2 interact and support the idea that these isoforms may form hetero- and homo-oligomers (Fig. 5j–m). Altogether, these data suggest that TGFβ signaling reduces the relative protein abundance of E isoforms as compared to that of the M/S isoforms of TCF7L2, which may lead to increase in M/S isoform-containing TCF7L2 protein complexes, which may facilitate EMT-like responses.

**Fig. 6:**
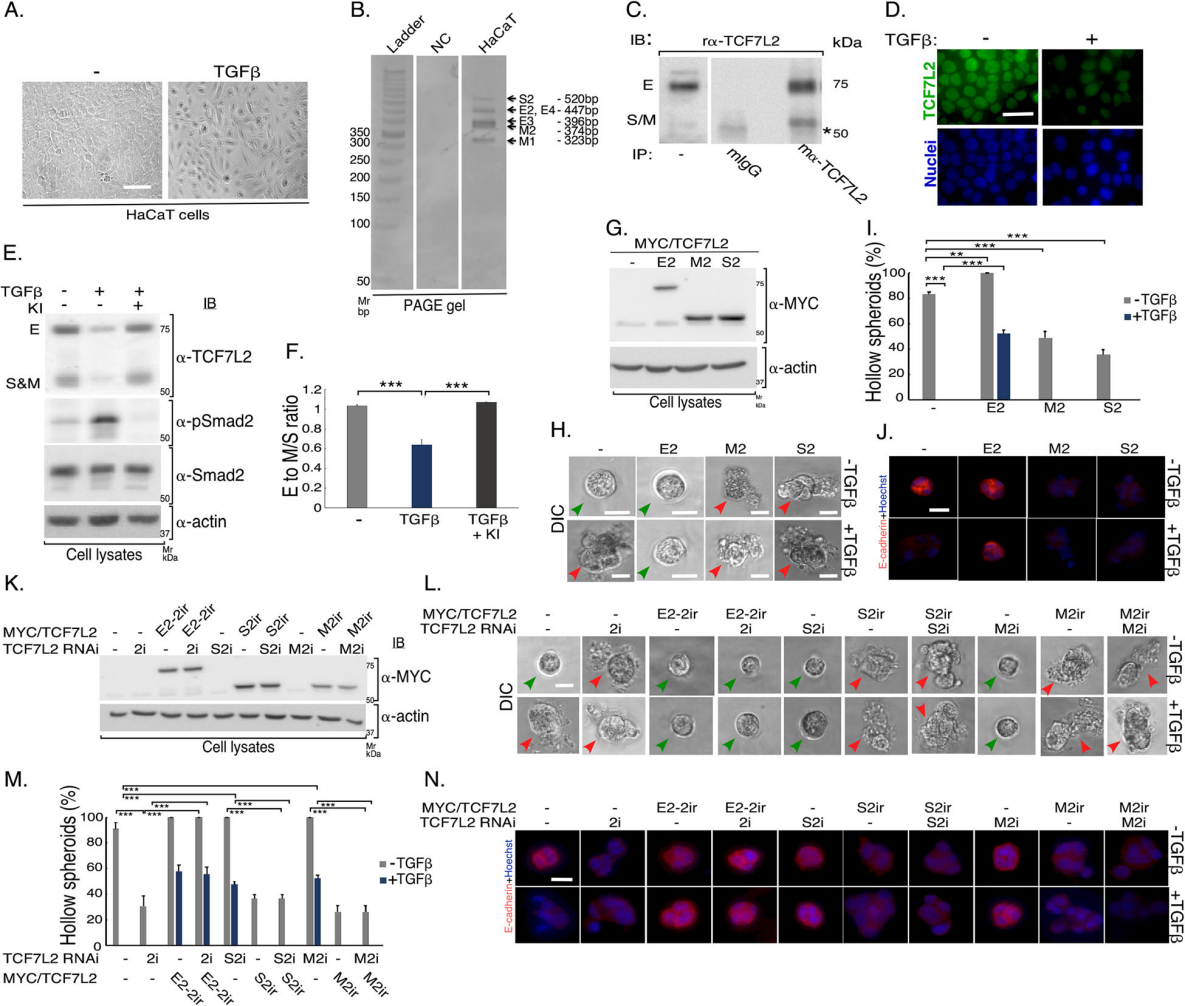
TGFβ-TCF7L2 isoform-specific signaling regulates EMT in three-dimensional skin epithelial cell-derived organoids. **a** Representative DIC scans of subconfluent monolayers of the human skin keratinocytes HaCaT cells left untreated or incubated with 100 pM TGFβ for 48 h. The scans are from one of three biological replicates of the experiment. Cuboidal-shaped HaCaT cells undergo EMT to form elongated spindle-shaped mesenchymal cell morphology. **b** Polyacrylamide gel resolved and stained C-variant TCF7L2 mRNA fragments amplified from HaCaT cDNA using S10f and S17r oligonucleotides (Table 1). **c** Lysates of HaCaT cells were subjected to immunoprecipitation (IP) using a mouse IgG or a mouse antibody recognizing the N-terminus of TCF7L2 followed by immunoblotting of the immunocomplexes and total lysates with a rabbit anti-TCF7L2 antibody (see Fig. S1A). TCF7L2 protein species with apparent molecular mass of ~75 kDa (E isoforms) and 60 (M/S isoforms) kDa were detected in the α-TCF7L2 immunoblotting of α-TCF7L2-immunocomplexes and lysates. **d** Representative fluorescence microscopy scans of TCF7L2- (TCF7L2 antibody, green) and nuclear-(Hoechst 33258, blue) stained formalin-fixed subconfluent monolayers of HaCaT cells left untreated or incubated with 100 pM TGFβ for 48 h. TGFβ led to approximately 80% reduction in total TCF7L2 isoforms in HaCaT cells undergoing EMT. The scans are from one of two biological replicates of the experiment. **e**–**f** TGFβ reduces the protein ratio of TCF7L2E to TCF7L2M/S in HaCaT cells. (**e**) TCF7L2, pSmad2, Smad2/3 and actin immunoblotting of lysates of HaCaT cells incubated without or with 100 pM TGFβ, alone or together with 10 µM KI for 48 h. **f** Bar graph mean (±SEM) protein ratio of E to M/S (*Y*-axis) versus incubation (*X*-axis) for experiments as described and assessed in 6**e** from three biological replicates of the experiment (ANOVA: ***P ≤ 0.001). **g**–**j** TCF7L2E2 suppresses, while TCF7L2M2/S2 promotes EMT induction in three-dimensional skin epithelial cell-derived spheroids. **g** Lysates of HaCaT cells transfected with the vector control or a plasmid-containing cDNA encoding MYC/TCF7L2E2 (E2), MYC/TCF7L2M2 (M2), or MYC/TCF7L2S2 (S2), were subjected to immunoblotting with MYC or actin antibody, with the latter used as loading control. **h** Representative DIC images of 8-day-old untreated or TGFβ-stimulated 3D-spheroids derived from HaCaT cells transfected as described in 6**g**. Green and red arrowheads indicate hollow center and filled/deformed spheroids, respectively. Scale bar indicates 50 μm. **i** Bar graph of average percent (±SEM) of hollow acinar untreated or TGFβ-stimulated 8-day-old spheroids derived from HaCaT cells transfected as in 6**g** from three biological replicates of the experiment, including the one shown in 6H (ANOVA: **P ≤ 0.01, ***P ≤ 0.001). TCF7L2E2 suppressed the ability of TGFβ to promote spheroid filling of HaCaT cell-derived spheroids. TCF7L2M2/S2 promoted spheroid filling even in the absence of TGFβ. **j** Representative fluorescence microscopy scans of E-cadherin- (E-cadherin antibody, red) and nuclear- (Hoechst 33258, blue) stained formalin-fixed untreated or TGFβ-stimulated 8-day-old 3D-spheroids derived from HaCaT cells transfected and subjected to three-dimensional culture as described in 6G-6I. Scale bar indicates 50 μm. TCF7L2E2 suppressed TGFβ‘s ability to decrease the protein abundance and/or mislocalization of E-cadherin, while TCF7L2M2/S2 promoted loss and mislocalization of E-cadherin even in the absence of TGFβ. **k**–**n** Endogenous TCF7L2 act in an isoform-dependent manner to regulate EMT in 3D-skin epithelial cell-derived spheroids. **k** Lysates of HaCaT cells transfected with a pU6 RNAi vector control plasmid (−), or one expressing the pan TCF7L2-2i (2i), or the isoform-specific TCF7L2S2i (S2i) or TCF7L2M2i (M2i) shRNAs, together with a vector control (−) or a plasmid expressing a MYC/TCF7L2 E2-2ir, S2-ir, or M2ir that is resistant to knockdown by TCF7L2- 2i, -S2i, or -M2i, respectively, were subjected to anti-MYC and anti-actin immunoblotting, with the latter used as loading control. **l** Representative DIC images of 8-day old untreated or TGFβ-stimulated 3D-spheroids derived from HaCaT cells transfected as described in 6**k**. Green and red arrowheads indicate hollow center and filled/deformed spheroids, respectively. Scale bar indicates 50 μm. **m** Bar graph of average percent (±SEM) of hollow- lumen untreated or TGFβ-stimulated 8-day-old 3D-spheroids derived from HaCaT cells transfected as described in 6**k** from three biological replicates of the experiment, including the one shown in 6**l** (ANOVA: ***P ≤ 0.001). Knockdown of endogenous TCF7L2 by TCF7L2-2i promoted hollow-lumen filling of spheroids even in the absence of TGFβ, which was reversed by TCF7L2E2-2ir. TCF7L2-S2i, and TCF7L2-M2i reduced TGFβ-induced filling of the hollow-lumen spheroids, which were reversed by the expression of the proteins TCF7L2S2-S2ir and TCF7L2M2-M2ir, respectively. **n** Representative fluorescence microscopy scans of E-cadherin- (E-cadherin antibody, red) and nuclear- (Hoechst 33258, blue) stained formalin-fixed untreated or TGFβ-stimulated of 8-day old 3D-spheroids derived from HaCaT cells transfected as described and subjected to three-dimensional culture in 6K-6M. Scale bar indicates 50 μm. Endogenous TCF7L2 knockdown by TCF7L2-2i reduced the protein abundance and led to mislocalization of E-cadherin even in the absence of TGFβ, which were reversed by TCF7L2E2-2ir. TCF7L2 knockdown by TCF7L2S2i and TCF7L2M2i suppressed the ability of TGFβ to reduce and mislocalize E-cadherin, which were reversed by the rescue proteins TCF7L2S2-S2ir and TCF7L2M2-M2ir, respectively.

We next asked if TCF7L2 might be regulated by TGFβ signaling in other epithelial cells besides NMuMG cells. Keratinocytes represent a specialized skin epithelial tissue-derived cell type. The human epidermal HaCaT keratinocytes is a widely used cellular model in studies of TGFβ-signaling and responses including EMT^33–35^. In our analyses, TGFβ promoted a cuboidal to elongated phenotype change in monolayer cultures of HaCaT cells (Fig. 6a). RT-PCR and immunoblotting analyses demonstrated that the TCF7L2 E and S/M isoforms are expressed in HaCaT cells (Fig. 6b, c). Immunocytochemistry analyses revealed that TCF7L2 is predominantly nuclear, and that TGFβ drastically reduced TCF7L2 in cells undergoing EMT (Fig. 6d). Similar to NMuMG cells, immunoblotting analyses demonstrated that TGFβ reduced the protein abundance of the TCF7L2 C-variant isoforms such that there was a significant reduction of the E to S/M isoform ratio (Fig. 6e, f). Next, we established 3D cultures in which HaCaT cells organized into hollow-centered spherical multicellular structures, which were disrupted upon TGFβ treatment, suggestive of induction of EMT-like effects (Figs. 6h, i and S7A). Consistently, TGFβ promoted mislocalization and reduction in the protein abundance of E-cadherin in the 3D-HaCaT-derived spheroids (Fig. 6j). Expression of TCF7LE2 isoform suppressed, while that of S2 and M2, promoted EMT-like behavior in the HaCaT-derived spheroids, as indicated by filling and deformation, and loss/mislocalization of E-cadherin in HaCaT cell-derived spheroids (Figs. 6g–j and S7A). Knockdown of endogenous TCF7L2 promoted EMT-like characteristics, an effect that was reversed by expression of shRNA2-resistant rescue form of TCF7L2E2 (Figs. 6k–n and S7B). In contrast, knockdown of endogenous M2 or S2 TCF7L2 suppressed the ability of TGFβ to induce EMT-like changes in HaCaT cell-derived spheroids, which was reversed by respective RNAi rescue TCF7L2 isoform protein (Figs. 6k–n and S7B). Collectively, our data suggest that the regulation of TCF7L2 by TGFβ signaling plays a critical role in EMT induction in epithelial cells.

### TCF7L2 acts via TGFβ signaling to regulate EMT and organoid morphology

**Fig. 7:**
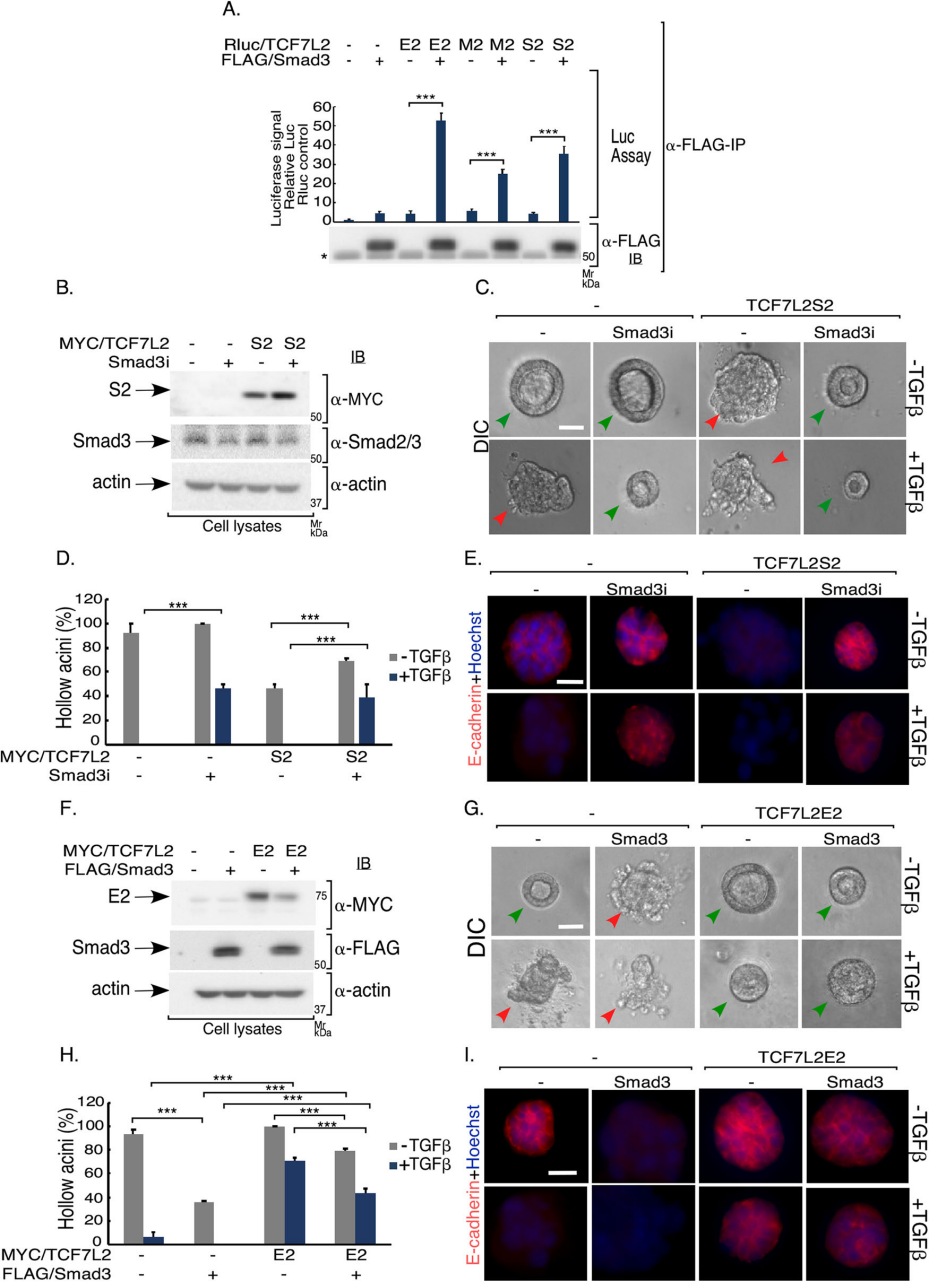
TGFβ-Smad3-TCF7L2 isoform-specific signaling regulates EMT and acini morphology. **a** Lysates of 293 T cells cotransfected with a plasmid-containing cDNA encoding the Renilla luciferase protein alone (−) or in fusion with TCF7L2 E2, M2, or S2, together with a vector control or a plasmid expressing FLAG/Smad3, were Smad3 immunoprecipitated (α-FLAG IP) followed by luciferase assay on 90% of the immunocomplexes, with the rest subjected to Smad3 immunoblotting (anti-FLAG IB). 10% of each lysate was subjected to luciferase as a measure of relative input. Upper panel—bar graph shows average (±SEM) input-normalized Smad3 immunocomplexes-containing luciferase values from ten biological replicates of the experiment (ANOVA: ***P ≤ 0.001). Lower panel—Smad3 level in the Smad3 immunocomplexes from a representative of ten biological replicates of the experiment, assessed as in 7**a**. Smad3 interacts with the TCF7L2 C-variants. **b**–**e** Endogenous Smad3 promotes TCF7L2S2-induced EMT of 3D-NMuMG cell-derived organoids. **b** Lysates of NMuMG cells transfected with vector control or MYC/TCF7L2S2 with a pU6 RNAi control vector (−) or one expressing Smad3 shRNAs (Smad3i), were subjected to MYC, Smad2/3, or actin immunoblotting, with the latter used as loading control. **c** Representative DIC images of 8-day old untreated or TGFβ-stimulated 3D-organoids derived from NMuMG cells transfected as described in 7**b**. Green and red arrowheads indicate hollow center and filled/deformed acini, respectively. Scale bar indicates 50 μm. **d** Bar graph of average percent (±SEM) of hollow acinar untreated or TGFβ-stimulated 8-day-old 3D-organoids derived from NMuMG cells transfected as in 7**b** from three biological replicates of the experiment, including the one shown in 7**c** (ANOVA: ***P ≤ 0.001). **e** Representative fluorescence microscopy scans of E-cadherin- (E-cadherin antibody, red) and nuclear- (Hoechst 33258, blue) stained formalin-fixed untreated or TGFβ-treated 8-day old 3D-organoids derived from NMuMG cells transfected and subjected to three-dimensional culture as described and shown in 7**b** and 7**c**. Knockdown of endogenous Smad3 suppressed the ability of TCF7L2S2 to induce EMT. **f–i** TCF7LE2 and Smad3 functionally interact to regulate EMT. **f** Lysates of NMuMG cells transfected with vector control or a MYC/TCF7L2E2 expressing plasmid, in absence or presence of FLAG/Smad3, were subjected to MYC, FLAG, or actin immunoblotting, with the latter used as a loading control. **g** Representative DIC images of untreated or TGFβ-stimulated 8-day-old 3D-organoids derived from NMuMG cells transfected as described in 7**f**. Green and red arrowheads indicate hollow center and filled/deformed acini, respectively. Scale bar indicates 50 μm. **h** Bar graph of average percent (±SEM) of hollow acinar untreated or TGFβ-stimulated 8-day-old 3D-organoids derived from NMuMG cells transfected as in 7**f** from three biological replicates of the experiment, including the one shown in 7**g** (ANOVA: ***P ≤ 0.001). **i** Representative fluorescence microscopy scans of E-cadherin- (E-cadherin antibody, red) and nuclear- (Hoechst 33258, blue) stained formalin-fixed untreated or TGFβ-stimulated 8-day-old 3D-organoids derived from NMuMG cells transfected and subjected to three-dimensional culture as described and shown in 7**f** and 7**g**. Scale bar indicates 50 μm.

TGFβ activation of the signaling protein Smad3 mediates EMT induction in the 3D-epithelial cell-derived acini^27^. We found that all C-variant isoforms of TCF7L2 associate with Smad3 in co-IP analyses (Fig. 7a). In other studies, we confirmed that knockdown of endogenous Smad3 reversed TGFβ-induced EMT in 3D-NMuMG cell-derived acini (Figs. 7b–e and S8A). Importantly, Smad3 knockdown also reversed the ability of expressed TCF7L2S2 isoform to promote EMT, suggesting that TCF7L2S2 isoform may act upstream to promote the ability of Smad3 to induce EMT (Figs. 7b–e and S8A). On the other hand, EMT-like effects promoted by Smad3 expression were partially overcome by expression of TCF7L2E2 (Figs. 7f–i and S8B). These data reveal that the isoform-specific functions of TCF7L2 in EMT-like effect in organoids are mediated by TGFβ-Smad3 signaling.

**Fig. 8:**
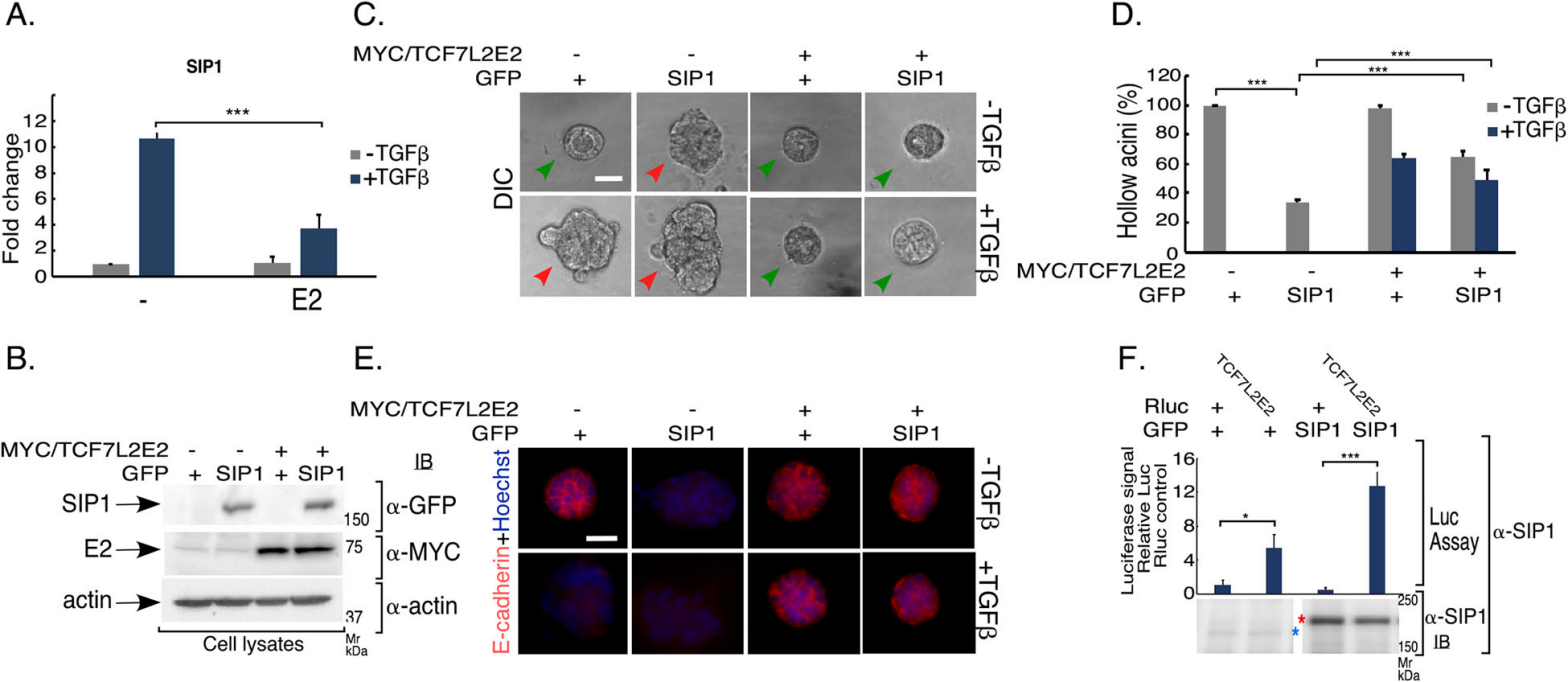
SIP1/Zeb2 contributes to TCF7L2E2 regulation of EMT. **a** TCF7L2E2 expression suppresses TGFβ-induced SIP1/Zeb2 mRNA expression. cDNA derived from untreated or TGFβ-stimulated vector control or TCF7L2E2-stably expressing NMuMG cells was subjected to SIP1/Zeb2 and GAPDH real time PCR assessment, with two technical repeats for each measurement per biological replicate of the experiment. The values from technical repeated were averaged for each measurement per biological repeat. Bar graph shows average (±SEM) of GAPDH-normalized SIP1/Zeb2 mRNA in untreated or TGFβ-stimulated control and TCF7L2E2 expressing NMuMG cells relative to that in the untreated control from seven biological replicates of the experiment, (ANOVA: ***P ≤ 0.001). **b**–**e** TCF7L2E2 -SIP1/Zeb2 interplay and EMT control. **b** Lysates of NMuMG cells transfected with vector control (−) or MYC/TCF7L2E2 (+) together with a plasmid expressing the green fluorescent protein (GFP) alone (+) or in fusion with SIP1, were subjected to MYC, GFP, or actin immunoblotting, with the latter used as loading control. **c** Representative DIC images of 8-day-old untreated or TGFβ-stimulated 3D-organoids derived from NMuMG cells transfected as described in 8**b**. Green and red arrowheads indicate hollow center and filled/deformed acini, respectively. Scale bar indicates 50 μm. **d** Bar graph of average percent (±SEM) of hollow acinar untreated or TGFβ-stimulated 8-day-old 3D-organoids derived from NMuMG cells transfected as in 8**b** from five biological replicates of the experiment, including the one shown in 8**c** (ANOVA: ***P ≤ 0.001). **e** Representative fluorescence microscopy scans of E-cadherin- (E-cadherin antibody, red) and nuclear- (Hoechst 33258, blue) stained formalin-fixed untreated or TGFβ-stimulated 8-day-old 3D-organoids derived from NMuMG cells transfected and subjected to three-dimensional culture as described and shown in 8**b** and 8**c**. Scale bar indicates 50 μm. **f** Lysates of 293 T cells transfected with a plasmid-containing cDNA encoding the Renilla luciferase protein (Rluc) alone (+) or in fusion with TCF7L2E2 together with a plasmid expressing the GFP alone (+) or in fusion with SIP1, were immunoprecipitated with an anti-SIP1 or IgG antibody followed by luciferase assay and SIP1 immunoblotting of 90% and 10%, respectively, of the immunocomplexes. For each transfection, 10% of the lysates was subjected to luciferase assays as a measure of relative input. Upper panel—bar graph shows average (±SEM) input-normalized luciferase values in the IgG-subtracted SIP1 immunocomplexes from four biological replicates of the experiment (ANOVA: *P ≤ 0.05, ***P ≤ 0.001). Lower panel—SIP1 immunoblot of the SIP1 immunocomplexes from of a representative of the four biological replicates of the experiment. Blue asterisk and red asterisk indicate endogenous and expressed SIP1 protein, respectively. SIP1 and TCF7LE2 associate.

The transcriptional regulator SIP1 is induced by TGFβ and is critical for induction of EMT, including by repressing E-cadherin. Expression of TCF7L2E2 downregulated TGFβ-induced SIP1 mRNA expression in NMuMG cells (Fig. 8a). We, therefore, tested if forced expression of SIP1 opposes TCF7L2E2 to suppress TGFβ-induced EMT. Expression of SIP1 promoted EMT in NMuMG cell-derived acini even in the absence of TGFβ (Figs. 8b–e and S9). However, TCF7L2E2 expression suppressed SIP1-induced EMT-like behavior (Figs. 8b–e and S9), suggesting that TCF7L2 may regulate EMT by downregulating SIP1 mRNA levels and suppressing the ability of the protein SIP1 to regulate EMT-gene expression. Supporting the latter idea, we found that SIP1 and TCF7L2E2 co-IP (Fig. 8f). Together, these data suggest that TCF7L2E2 may suppress the ability of Smad3 and SIP1 to regulate expression of EMT signature genes at different levels.

Collectively, our study reveals that TCF7L2E and TCF7L2M/S isoforms suppress and promote, respectively, TGFβ-induced EMT-like effects in 3D-epithelial-derived organoids. Consistently, we find that TGFβ reduces the E to M/S protein isoform proportion in cells undergoing EMT. Mechanistically, our studies suggest that the TCF7L2E isoforms may suppress EMT at the level TGFβ-Smad-SIP1 signaling axis.

## Discussion

In this study, we have discovered novel isoform-specific functions for the major transcription factor TCF7L2 and identify novel links of TCF7L2 with TGFβ signaling in the control of EMT and epithelial tissue morphogenesis. Our data reveal that the C-variants TCF7L2 isoforms exert opposing functions in EMT regulation. Whereas, TCF7L2E isoforms suppress, the TCF7L2S/M isoforms promote EMT with consequences for 3D-epithelial cell organoid’s morphogenesis. We have also found that β-catenin signaling may not be needed for the C-variant isoforms of TCF7L2 to regulate TGFβ-induced EMT. In mechanistic studies, TGFβ signaling reduces the proportion of the TCF7L2E to TCF7L2S/M abundance. We have found that TCF7L2E associates with Smad3 and the EMT driver SIP1 to suppress EMT. The TGFβ-Smad3-TCF7L2 isoform-specific signaling pathway uncovered here should advance our understanding of mechanisms that regulate development and diseases.

The opposing roles of the TCF7L2 isoforms in EMT unraveled in this study add key insights into the biological versatility of this transcription factor. TCF7L2 is implicated in regulating embryonic brain development and maintenance of intestinal and breast-derived epithelial tissues^6,8,12^^,17^. Deregulation of TCF7L2 may be involved in diseases including multiple sclerosis, type II diabetes, stroke, and cancer^6^^,15^^,36–44^. TCF7L2 has been suggested to act as an oncogenic or tumor suppressor in colorectal and breast carcinomas^15,40–42,45^. As EMT is key for proper tissue and organ development, our findings provide an important basis for future studies to characterize isoform-specific TCF7L2 roles in development and disease.

Our structure-function analyses point to a C-terminal-containing region to be essential for the TCF7L2E isoforms to maintain the epithelial phenotype in the face of TGFβ-induced EMT. Several domains/motifs may contribute to the ability TCF7L2E isoforms to suppress EMT as reflected by alignments of the divergent C-termini of TCF7L2, M and S protein isoforms. In particular, a full C-clamp domain, the CtBP-binding motifs, alone or together might contribute to the ability of TCF7L2E isoforms to suppress EMT, as they are absent in the M and S isoforms. As the E isoforms of TCF7 and TCF7L1 harbor a C-clamp domain and CtBP-binding motifs, respectively, it will be interesting to determine if corresponding isoforms of these TCF/LEF members display differential biological effects including in EMT.

TCF7L2 has been largely suggested to mediate the canonical Wnt-β-catenin signaling to regulate cellular processes^4,5^. However, recent evidence suggests that TCF7L2 may also act in Wnt signaling-independent manner, for example in a bone morphogenetic protein (BMP) signaling-dependent manner to promote oligodendrocyte differentiation^4^. Our findings that the β-catenin-domain deletion does not influence the isoform-specific TCF7L2-mediated responses, may provide further support for the idea that Wnt-β-catenin signaling may not regulate TCF7L2 role in EMT, with potential significance to in vivo processes. Identification of EMT-gene signature candidates and their responsive elements that are differentially regulated by the E vs S/M TCF7L2 isoforms can address whether regulation of β-catenin-responsive elements in these genes contributes to the isoform-specific function of TCF7L2 in EMT.

Our novel finding that TCF7L2 functions in TGFβ signaling and potentially independently of β-catenin signaling has raised several key questions. What are the implications for other TGFβ responses? Might TCF7L2 be relevant for other TGFβ outcomes besides EMT-like effects? Likewise, might TCF7L2 functions in other biological responses be subject to TGFβ signaling regulation?

Our mechanistic studies provide a model in which TCF7L2E/S/M associates with Smad3 potentially via a common domain/motif including on promoters of TGFβ-responsive genes with implications for EMT induction^5^^,46,47^. Supporting this idea is our finding that TCF7L2E2 suppresses TGFβ-induced expression of the major EMT driver SIP1^27,48^. Further, our novel finding that TCF7L2 isoforms form homo-, and hetero-oligomers suggests the possibility that these complexes may have a role in regulating EMT-like responses, and thus adds complexity to the mechanisms in control of this key process.

In conclusion, our data suggest a model where the cellular level of TCF7L2E isoforms controls the ability of cells to maintain an epithelial phenotype under condition of no/low TGFβ signaling, and where increased TGFβ signaling triggers events that reduces the proportion of TCF7L2E to M/S isoforms that leads to cells undergoing EMT. These findings add insight into the regulation of EMT-like effects including in development and diseases.

## Materials and methods

### Plasmids

pCMV5B, a cytomegalovirus (CMV)-based mammalian expression plasmid, containing cDNA encoding MYC-tagged mouse TCF7L2E2 with (MYC/TCF7L2E2ex4) or without (MYC/TCF7L2E2) exon 4 was generated using a conventional molecular biology cloning strategy as follows. DNase-treated TRIzol (Ambion, Life technologies)-NMuMG cells-extracted RNA was reverse transcribed using SuperScript II transcriptase (Invitrogen) and oligo- (dT)12-18 (Amersham Biosciences)^49^. The polyA-cDNA was subjected to PCR amplification of the TCF7L2E isoforms open reading frame using the forward E1f and reverse E17r-i oligonucleotides (Table 1), and subcloning into pCMV5B/MYC using convenient restriction sites. To generate pCMV5B/MYC/TCF7L2E3, E4, M1, M2, or S2, NMuMG-derived polyA-cDNA was subjected to PCR amplification using as forward E12f and reverse E17r-ii oligonucleotides (Table 1) corresponding to exons 12, and 17, respectively, followed by 10% polyacrylamide gel-separation, purification (Qiagen gel extraction Kit (Qiagen)), sequencing, and subcloning of respective C-variant fragment into restriction endonuclease digested pCMV5B/MYC/TCF7L2E2 replacing the E2- C-variant fragment. To generate a β-catenin binding domain (ΔN)-deleted TCF7L2 expression vector, TCF7L2 was subjected to PCR amplification by forward E2f and reverse E6r oligonucleotides (Table 1), and subcloned into a digested pCMV5B/MYC/TCF7L2E2, M2 or S2 plasmid using convenient restriction sites. Hemagglutinin (HA)- tagged TCF7L2E2, HA/TCF7L2M2 or HA/TCF7L2S2 were generated by subcloning the corresponding TCF7L2 isoform into pCMV5B/HA expression vector. To establish NMuMG stable cell lines, a pCAGIP vector was employed to generate constructs containing cDNA encoding MYC/TCF7L2E2, with the cDNA encoding TCF7L2 and the puromycin resistance marker driven by the upstream promoter to generate a bicistronic transcript with Internal Ribosomal Entry Site (IRES) leading to express TCF7L2 and resistance marker proteins^50^. Expression plasmids containing cDNA encoding U6 promoter-based control RNAi vector, coexpressing enhanced green fluorescent protein (EGFP) under the control of a CMV promoter, was used to generate TCF7L2 pan RNAi plasmids RNA-1i and RNA-2i containing double stranded DNA encoding short hairpin (sh) RNA1, shRNA2 to target nucleotides common to all TCF7L2 isoforms (Table 2). The RNAi plasmids RNA-M2i and RNA-S2i encoding the expression of TCF7L2 shRNA-M2i and shRNA-S2i were generated to selectively knockdown the TCF7L2M2 and TCF7L2S2 isoforms, respectively (Table 2)^50^^,51^. Expression RNAi rescue constructs were generated encoding the following RNAi-resistant TCF7L2 protein isoform: E2, M2, or S2 RNA-1i rescue (E2-1ir, M2-1ir, or S2-1ir), E2 RNA-2i-rescue (E2-2ir), M2 RNA-M2i rescue (M2ir), and S2 RNA-S2i rescue (S2ir), by changing the TCF7L2 cDNA at the nucleotide level, while keeping the amino acid sequence intact, in the region targeted by the specific TCF7L2 RNAi plasmid. Plasmids were validated by DNA sequence analyses (University of Calgary Core Sequencing Facility, Calgary, AB, Canada). FLAG/Smad3 and green fluorescent protein (GFP)/Smad Interacting Protein 1 (SIP1) have been described^52^.

**Table 1:** Oligonucleotide related information.

List and name	sequence (5’ →3’)
E1f	GGCGTCGACAACCGCAGCTGAACGGCGGTGG
E17r-i	CCGTCTAGACTATTCTAAAGACTTGGTCACCAGAGACAG
E12f	ACAAGCAGCCGGGGGAAAC
E17r-ii	AGCCTAGCAGATGCGGTGAG
E2f	GGCGTCGACAAACGAATCAAAACAGCTCCTCCG
E6r	TTCCTGTTTTGGGGTCTACG
E10f	CTGAGTGCACGTTGAAAGAAAG
q-TCF7L2-f	CCGAATGCACATTGAAAGAGAG
q-TCF7L2-r	GGGCCAGCTCGTAGTATTT
q-SIP1-f	CTCATTCTGGGTCCTACAGTTC
q-SIP1-r	GGGAAGAACCCGTCTTGATATT
q-E-cad-f	CTGCTGCTCCTACTGTTTCTAC
q-E-cad-r	TCTTCTTCTCCACCTCCTTCT
q-GAP-f	TCAACAGCAACTCCCACTCTTCCA
q-GAP-r	ACCCTGTTGCTGTAGCCGTATTCA

**Table 2:** RNAi plasmids related information.

Name	target sequence in mouse (5’ →3’)	No. of mismatches in human
TCF7L2-1i	GCACACATCGTTTCGAACAAA	2
TCF7L2-2i	GTACAGCAATGAACACTTCA	0
TCF7L2-M2i	CCTCCGATCACAGGAGAAA	1
TCF7L2-S2i	CCCTGCAGATGCAAATACGC	2
Smad3i	GTGCGAGAAGGCGGTCAAGAG	0

### Cell lines and transfections

The NAMRU mouse mammary gland epithelial cells (NMuMG) (American Type Culture Collection (ATCC), Cat # CRL-1636) were maintained in Dulbecco’s modified Eagle’s medium with high glucose and L-glutamine (Invitrogen), supplemented with 10 mg/ml insulin (Invitrogen, cat # 12585-014) and 10% fetal bovine serum. A human adult keratinocyte cell line (HaCaT)^53^^,54^ were cultured in Dulbecco’s modified Eagle’s medium containing 10% FBS (FBS, Gibco, Cat # 12483-020). Human embryonic kidney epithelial 293 T cells were cultured in Dulbecco’s modified Eagle’s medium with high glucose and L-glutamine supplemented with 10% fetal bovine serum. All the cells were confirmed to be free of pathogenic Mycoplasma strains by a PCR-ELISA kit (Roche, Cat # 11663925910). The 293 T cells were transiently transfected using the calcium phosphate precipitation method, and NMuMG and HaCaT cells were transfected using Lipofectamine LTX Plus reagents (Thermo Fisher Scientific, Cat # 15338100).

### Co-immunoprecipitation and immunoblotting

Non-transfected or transfected cells of monolayer cultures were washed with 1X phosphate-buffered saline (PBS), followed by incubation with 1X TNE (pH 7.3) lysis buffer (50 mM Tris, 150 mM NaCl, and 1 mM EDTA and 0.5% Triton X-100), containing trypsin inhibitors, protease inhibitors, pepstatin A and Phenyl methane sulfonyl Fluoride (PMSF) as protease inhibitors and sodium fluoride, sodium pyrophosphate and sodium vanadate as phosphatase inhibitors for 20 min at 4 °C. Cellular extracts were collected into 1.75 ml Eppendorf tubes and centrifuged at 14,000 rpm for 10 min at 4 °C in Sigma centrifuge. Approximately 90% of the cell lysates’ supernatants were collected and subjected to mouse TCF7L2 (Millipore, cat # 6H5-3) or control IgG (Santa-Cruz, cat # sc-2025) or rabbit TCF7L2 (cell signaling, cat # C48H11) or control IgG (Santa-Cruz, cat # sc-2027), or mouse anti-HA (Biolegend, Cat # 901515) immunoprecipitation at 4 °C. The remaining 10% of the supernatants were used for protein quantification (Bradford protein assay/spectroscopic analysis procedure, Biorad Cat # 500-0006) and determining the abundance of specific proteins by immunoblotting. Proteins in the supernatants and immunoprecipitations were resolved by sodium dodecyl sulfate- polyacrylamide gel electrophoresis (SDS-PAGE), followed by transfer to nitrocellulose membranes (Biorad, Cat # 1620115), and probing using mouse anti-TCF7L2 (Millipore, Cat #6H5-3), rabbit anti-TCF7L2 (Cell Signaling technology, Cat # C48H11), mouse anti-HA (Biolegend, Cat # 901515), mouse anti-MYC (Santa-Cruz, Cat # sc-40), mouse anti-FLAG (Sigma, Cat # F1804-5MG), or mouse anti-actin (Santa-Cruz, Cat # sc-47778) as primary antibodies and HRP-conjugated goat anti-mouse or donkey anti-rabbit IgG (Jackson Laboratories) as secondary antibodies, followed by incubation in Enhanced Chemi-luminescence (ECL) (Millipore Cat # WBLUR0500) reagent. Chemi-luminescence light signal detection was done using VersaDoc 5000 Imaging system and densitometry was performed using Quantity One software (Bio-Rad Laboratories).

Renilla luciferase (Rluc)-based assessment of co-immunoprecipitating Rluc only (control) or Rluc/TCF7L2 protein involved subjecting 90% of a given immunoprecipitation resuspended in 50 ul 0.1% Triton X-100-containing TNE buffer to luciferase assay (Luc assay). The remaining 10% of the immunocomplexes were subjected to immunoblotting to confirm the presence of an immunoprecipitated protein. For each transfection per biological replicate, 10% of the lysate was subjected to luciferase measurement as the “input” for the Rluc or Rluc/TCF7L2 protein in a given immunoprecipitation. Renilla luciferase values were obtained using the Renilla luciferase kit (Promega kit) coupled to the Orion II luminometer (Titertek–Berthold) detection system. For each transfection in a biological replicate per experiment, input-normalized coimmunoprecipitated Rluc value was expressed relative to the biological replicate’s global average and subjected to statistical analysis, followed by expression relative to the corresponding value of the Rluc only control, and plotting as mean ± SEM of the biological replicates of given experiment as a bar graph.

In case of the lysing of three-dimensional cell-derived multicellular structures, cell cultures were subjected to vigorous up and down pipetted to help dislodge the colonies form the Matrigel-based ECM. Cell-Matrigel mixtures from several technical replicates per condition of a biological replicate were pooled in ice-cold serum-free DMEM followed by resuspension of the mixture using a pipet to isolate cellular aggregates from the Matrigel. The isolated cell suspensions were centrifuged at 1500 rpm for 5 min, and pellets were washed once with 1X PBS and lysed as described in the cell lysis procedure for monolayer cultures, and lysates were subjected to immunoblotting as described.

### Generation and analyses of three-dimensional epithelial cell-derived multicellular structures

Epithelial cells were trypsinized and used for the generation of three-dimensional cultures^26,27^. Seventy-five microliter of ice-cold 30% growth factor-reduced Matrigel (Corning, Cat # 354230) solution (3 mg/ml final concentration) in growth medium containing antibiotics and antimitotic agents (complete medium) was added to a low-attachment well of an 8-well chamber slides (Millipore, Cat # PEZGS0816). The chamber slides were kept at 37 °C in a 5% CO2 incubator for 1 h to allow the setting of Matrigel cushions. Next, a mixture of ~800 epithelial cells in 75 ul of ice-cold 50% growth factor-reduced Matrigel solution (5 mg/ml final concentration of the Matrigel) in complete medium was carefully layered on top of the Matrigel cushion in each well of the 8-well chambers. 100 μL of complete medium was layered on top 1 h later, and the cultures were kept in a controlled 5% CO2 humidified incubator at 37 °C. The next day, the three-dimensional cell cultures were incubated in the absence or presence of 20 pM TGFβ, or 10 µM of the TGFβ type I receptor ser/thr kinase small molecule inhibitor SB431542 (KI for short-Figs. 2 and 3) in a 5% CO2 humidified incubator at 37 °C. 8-day-old three-dimensional epithelial cell-derived multicellular structures or “organoids”, a total of ~30–40, were visualized by light microscopy and qualitatively assessed for (a) hollow vs filled acini and (b) organized vs deformed morphology, before capturing the images of 8–10 randomly selected organoids using Differential Interference Contrast (DIC) microscopy with an inverted microscope at a ×20 objective lens for presentation and quantitative analyses (Olympus IX70, Olympus Canada Inc) (see below). The multicellular structures were then fixed with 4% formaldehyde, followed by permeabilization using 0.5% cold Triton X-100 solution and blocking using 5% BSA in phosphate-buffered saline. The multicellular structures or organoids were subjected to immunocytochemical analyses using a rabbit E-cadherin antibody (Cell Signaling Technology, Cat # 3195) as the primary antibody and Cy5-conjugated anti-rabbit IgG (Jackson Lab, Burlington, ON, Canada) as the secondary antibody along with the DNA dye bisbenzimide (Hoechst 33258, Invitrogen) to visualize nuclei. Immunofluorescent images of the multicellular colonies were captured using a fluorescent microscope with a ×40 objective lens (Olympus IX70, Olympus Canada Inc). Exposure times for E-cadherin and Hoechst-specific signals were kept constant in each biological replicate of a given experiment. For morphological analyses of the organoids imaged using DIC microscopy, for each experimental condition, the multicellular structures were scored for (1) presence or absence of hollow lumen, and (2) presence or absence of deformation including budding and invasive morphological characteristics. The data were plotted as bar graphs versus experimental condition and statistically analyzed as described in the figure legends and under the statistical analyses section.

### Indirect immunofluorescence

Eight-well chamber-slide-seeded epithelial NMuMG or HaCaT cells were subjected to indirect immunofluorescence as described^55^. Briefly, the epithelial cells were fixed with 4% formaldehyde (Thermo Fisher, Canada), permeabilized with 0.2% Triton-X-100, and blocked using 5% bovine serum albumin (BSA, Sigma) and 5% calf serum (VWR, Canada) in phosphate-buffered saline (PBS). Subcellular localization and abundance of TCF7L2 were determined by incubating NMuMG cells with a mouse anti-TCF7L2 antibody (Millipore, Cat # 6H5-3) followed by Cy2-conjugated anti-mouse IgG (Jackson Lab, Burlington, ON, Canada) using a well-established protocol^55^. Cells were incubated with the DNA fluorescent dye Hoechst 33342 (Invitrogen) to visualize their nuclei. Immunofluorescent images of cells were captured using a fluorescence microscope with a 40 X objective lens (Olympus IX70). For each experiment and each fluorophore, the exposure time was kept constant. The pixel signal intensity for TCF7L2 immunofluorescence for all cells in three to four fields for each treatment condition was quantified using ImageJ software (NIH, Bethesda). The percent change in the TCF7L2 pixel signal intensity by TGFβ as compared to the untreated cells was determined and expressed as mean (±SEM) from two independent biological replicates.

### RT- PCR

DNase-treated TRIzol (Gibco)-extracted RNA from NMuMG or HaCaT cells was reverse transcribed using SuperScript II transcriptase (Invitrogen) and oligo- (dT)12-18 (Amersham Biosciences)^26^. The polyA-cDNA was subjected to PCR amplification using Taq DNA polymerase (NEB, Cat # M0267L), together with the TCF7L2 forward primer E12f (NMuMG) or E10f (HaCaT) and the reverse conserved primer E17r-ii, with the two primer sets flanking a region between exons 12 and 17 or 10 and 17, respectively. (Table 1). The PCR products were resolved using 10% polyacrylamide gel electrophoresis, stained with redsafe dye at 1:20,000 (iNtRON biotechnology inc), and visualized and imaged using VersaDoc 5000 Imaging system to ascertain the C-terminal variants of TCF7L2 cells.

### Quantitative reverse transcription (RT)-PCR

RNA extracted from NMuMG cells using DNase-treated TRIzol (Gibco, Cat # 15596026) was reverse transcribed using SuperScript II transcriptase (Invitrogen, Cat # 18064014) and oligo- (dT)12-18 (Invitrogen, Cat # 18418012)^26^. The cDNAs were subjected to quantitative PCR of the following genes: TCF7L2: forward q-TCF7L2-f, reverse q-TCF7L2-r; SIP1: forward q-SIP1-f, reverse q-SIP1-r; and E-cadherin: forward q-E-cad-f, reverse q-E-cad-r (Table 1), and as a house-keeping gene glyceraldehyde-3-phosphate dehydrogenase (GAPDH): forward q-GAP-f, reverse q-GAP-r (Table 1), employed as an internal control, using a 2X Sybr Green Mix (BioRad, Cat # 1725272) and Rotor-Gene Thermocycler (Corbett Research). The specificity of the amplification products was confirmed using the melting curve method. Data were analyzed and expressed as described^26^^,56^. The 2^–∆∆**Ct**^ method was used to determine the GAPDH-normalized expression of a gene of interest and expressed relative to the experimental control condition. Each condition per biological replicate per experiment was repeated twice, and values averaged for obtaining the mean of the biological replicates.

### Statistical analyses

Number and type of replicates, i.e., biological versus technical repeats, are indicated in the legends of Figs. 1–8. The term “biological replication” in this study refers to the use of a distinct source of cultured cells for the various tested parameters per replicate for a given experiment. Thus, the replicates are performed in an independent manner. The term “technical replication” refers to subjecting the same sample to repeated measurement. Data from experiments carried out using biological replicates were subjected to statistical analysis by *t*-test or ANOVA followed by Student–Newman–Keuls test (InStat, San Diego, CA, USA). Values of *P* < 0.05 were considered statistically significant. Single, double and triple asterisks (*, **, and ***) denote significance at *P* ≤ 0.05, *P* ≤ 0.01, and *P* ≤ 0.001, respectively. Data are presented graphically as mean ± standard error of the mean (SEM) of biological replicates that were performed at least three independent times for a given experiment.

## Supplementary information

Legends for supplementary figure

Supplementary Figure 1

Supplementary Figure 2

Supplementary Figure 3

Supplementary Figure 4

Supplementary Figure 5

Supplementary Figure 6

Supplementary Figure 7

Supplementary Figure 8

Supplementary Figure 9
