# Supplementary material for: Regulation of epithelial-mesenchymal transition and organoid morphogenesis by a novel TGFβ-TCF7L2 isoform-specific signaling pathway
Source: Cell Death Dis. 2020 Aug 25;11(8):704. doi: 10.1038/s41419-020-02905-z (PMC7447769; doi:10.1038/s41419-020-02905-z)
Supplement: Supplementary file 1 — Legends for supplementary figure [file 41419_2020_2905_MOESM1_ESM.docx]

**Legends for Supplementary Figures**

**Figure S1. Characterization of TCF7L2 isoform expression in mammary epithelial cells**

(A) Schematic representations of TCF7L2 mRNA splicing and protein isoform structure and domains. TCF7L2 pre-mRNA, comprising 17 exons, undergoes alternative splicing leading to diverse TCF7L2 splice forms. Exons colored in orange are alternatively spliced exons, and those in light green are constitutively spliced exons. Exon 1 encodes β-catenin binding domain (green), exon 10-11 encode a high mobility group (HMG) domain (blue), exon 12 encodes nuclear localization sequence or NLS (brown). Splicing of exon 14 or 15 and part of exon 17 encodes C-clamp (red). A C-terminal binding protein (CtBP) domain is encoded by C-terminal part of exon17. Alternative splicing of exons 13,14,15 and 16 yields three C-variant isoforms termed: Extended (E), Medium (M) or Short (S), with E having, and M lacking the C-clamp and CtBP domains, and the S isoform only containing a partial C-clamp. Target regions of TCF7L2 antibodies used in this study are shown (red- mouse TCF7L2, 6H5-3 and black- rabbit TCF7L2, C48H11). Schematics of TCF7L2 isoforms E2, E3, E4, M1, M2 and S2 at the mRNA (left) and protein (right) levels relevant to this study are shown below the pre-mRNA. Relative region targeted by RNA1i, RNA2i, RNAM2i and RNAS2i plasmids are depicted as a red, yellow, orange, and green box, respectively, above the TCF7L2 mRNA. (B) Lysates of NMuMG cells were subjected to immunoprecipitation (IP) using an antibody recognizing the N-terminal or Mid-region of TCF7L2 followed by immunoblotting with the reciprocal TCF7L2 antibody. Both antibodies recognize protein species with apparent molecular mass of approximately 75kDa and 60kDa in immunocomplexes and the lysates (-). Asterix (*) indicates antibody's heavy chain band. (C) Approach used to PCR-amplify and subclone TCF7L2E2 into an expression vector (see methods). (D) The ~1.7 kb PCR product represents a mixture of E isoforms (without and with exon 4), including E2 which was first cloned. (E) A schematic of subcloning strategy to generate expression vectors of C-terminal variant TCF7L2. (F) Polyacrylamide-resolved and stained C-variant TCF7L2 mRNA fragments amplified from NMuMG cDNA as in E and used to generate the corresponding TCF7L2 isoforms.

**Figure S2- Related to Figure 1**

(A) Bar graph of average (±SEM) percent deformed 8-day old untreated or TGFβ-stimulated organoids derived from NMuMG cells transfected as in Figure 1A from three biological replicates of the experiment, including the one shown in Figure 1B (ANOVA: ***P ≤ 0.001, ns- not significant). Expression of TCF7L2E2, with or without exon 4, suppressed the ability of TGFβ to promote deformation of epithelial cell-derived organoids. (B) Bar graph of average (±SEM) percent deformed 8-day old untreated or TGFβ-stimulated organoids derived from NMuMG cells transfected as in Figure 1E from three biological replicates of the experiment, including the one shown in Figure 1F (ANOVA: ***P ≤ 0.001). Like E2, E3 or E4 expression suppressed TGFβ-induced organoids deformation, whereas S2, M1 or M2 expression promoted organoid deformation even in the absence of exogenous TGFβ. (C) Bar graph of average (±SEM) percent deformed of untreated or TGFβ-stimulated 8-day old 3D-organoids derived from NMuMG cells transfected as described in Figure 1I from three biological replicates of the experiment, including the one shown in Figure 1J (ANOVA: ***P ≤ 0.001). E2 and M2/S2, with or without exon 4, prevents and promotes, respectively, the deformation of organoids.

**Figure S3- Related to Figure 2**

(A) Bar graph of average percent (± SEM) of deformed untreated or TGFβ-stimulated 8-day old organoids derived from NMuMG cells transfected as in Figure 2A from three biological replicates of the experiment, including the one shown in figure 2B (ANOVA: ***P ≤ 0.001). (B) Bar graph of average percent (± SEM) of deformed vehicle-treated (-KI) or TGFβ signaling inhibitor-treated (+KI) 8-day old organoids derived from mammary epithelial cells transfected as described in Figure 2E from three biological replicates of the experiment, including the one shown in Figure 2F (ANOVA: **P ≤ 0.01,***P ≤ 0.001, ns- not significant). Inhibition of basal TGFβ signaling reversed the ability of TCF7L2S2 isoform to promote acini deformation.

**Figure S4- Related to Figure 3**

(A) Bar graph of average percent (± SEM) of deformed untreated, KI, or TGFβ-incubated 8-day old organoids derived from NMuMG cells transfected as described in Figure 3A from three biological replicates of the experiment, including the one shown in Figure 3C (ANOVA: **P ≤ 0.01, ***P ≤ 0.001). TCF7L2-1i and 2i promoted organoid deformation even in the absence of TGFβ, and which was reversed by TGFβ signaling inhibition (KI). (B) Bar graph of average percent (± SEM) of deformed untreated or TGFβ-incubated 8-day old 3D-organoids derived from NMuMG cells transfected as described in Figure 3F from four biological replicates of the experiment, including the one shown in Figure 3G (ANOVA: * P ≤ 0.05, **P ≤ 0.01, ***P ≤ 0.001). (C-G) *EMT induced by endogenous TCF7L2 knockdown is not affected by expression of pan RNAi-rescue TCF7L2M2 or TCF7L2S2 protein.* (C) Lysates of NMuMG cells transfected with pU6 RNAi vector control (-), or one expressing TCF7L2-1i, together with a control expression vector, or a plasmid containing cDNA encoding HA/TCF7L2M2 or HA/TCF7L2S2 that is sensitive (M2 or S2) or resistant (M2-1ir or S2-1ir) to TCF7L2-1i, were subjected to HA and actin immunoblotting, with the latter as a loading control. (D) Representative DIC images of untreated or TGFβ-stimulated 8-day old organoids derived from NMuMG cells transfected as described in S4C. (E) Bar graph of average percent (± SEM) of hollow acinar untreated or TGFβ-stimulated of 8-day old 3D-organoids derived from NMuMG cells transfected as described in S4C from two biological replicates of the experiment including those shown in S4D. (F) Bar graph of average percent (± SEM) of deformed untreated or TGFβ-stimulated 8-day old 3D-organoids derived from NMuMG cells transfected as described in S4C from two biological replicates of the experiment including those shown in S4D. (G) Representative fluorescence microscopy scans of E-cadherin- (E-cadherin antibody, red) and nuclear- (Hoechst 33258, blue) stained formalin-fixed 8-day old untreated, or TGFβ-stimulated 3D-organoids derived from NMuMG cells transfected and subjected to three-dimensional culture as described and shown in S4C and S4D

**Figure S5- Related to Figure 4**

(A) Bar graph of average percent (± SEM) of deformed untreated or TGFβ-stimulated 8-day old 3D-organoids derived from NMuMG cells transfected as described in Figure 4A from three biological replicates of the experiment, including the one shown in Figure 4B (ANOVA: **P ≤ 0.01, ***P ≤ 0.001). TCF7L2M2i reduced TGFβ-induced organoid deformation, which was reversed by expression of the RNAi rescue protein TCF7L2M2-M2ir. (B) Bar graph of average percent (± SEM) of deformed untreated or TGFβ-stimulated of 8-day old organoids (± SEM) derived from NMuMG cells transfected as described in Figure 4E from three biological replicates of the experiment, including the one shown in Figure 4F (ANOVA: ***P ≤ 0.001). TCF7L2S2i reduced TGFβ-induced organoids deformation, which was reversed by expression of the RNAi rescue protein TCF7L2S2-S2ir.

**Figure S6- Related to Figure 5**

(A and B) Bar graphs of average (±SEM) of relative GAPDH-normalized (A) SIP1/Zeb2 or (B) E-cadherin mRNA determined by qRT-PCR of cDNA derived from untreated or TGFβ-stimulated NMuMG cells from four biological replicates of the experiment (ANOVA: **P ≤ 0.01, ***P ≤ 0.001). For each biological replicate, cDNA was also subjected to TCF7L2 qRT-PCR as described and shown in Figure 5B. (C) SIP1/Zeb2, E-cadherin and actin immunoblotting of lysates of 48h-monolayer (2D) or 8-day organoid (3D) cultures derived from NMuMG cells, and left untreated or stimulated with TGFβ. Representative blots are shown from one of four biological replicates of the experiment as described and shown in Figure 5E. (D) Lysates of NMuMG cells transfected with increasing amount (0x, 1x, 10x, 100x) of HA/TCF7L2S2 (HA/S2) expressing plasmid in the absence or presence of a constant amount of a plasmid (100x) encoding MYC/TCF7L2E2 (MYC/E2), were subjected to immunoblotting with HA, MYC, or actin antibody, with the latter used as loading control. (E) Bar graph of average percent (± SEM) of deformed untreated or TGFβ-stimulated 8-day old 3D-organoids derived from NMuMG cells transfected as in S6D from three biological replicates of the experiment, including the one shown in Figure 5G (ANOVA: *P ≤ 0.05,**P ≤ 0.01,***P ≤ 0.001).

**Figure S7- Related to Figure 6**

(A) Bar graph of average percent (± SEM) of deformed untreated or TGFβ-stimulated 8-day old spheroids derived from HaCaT cells transfected as in Figure 6G from three biological replicates of the experiment, including the one shown in Figure 6H (ANOVA: ***P ≤ 0.001). (B) Bar graph of average percent (± SEM) of deformed untreated or TGFβ-stimulated 8-day old 3D-spheroids derived from HaCaT cells transfected as described in Figure 6K from three biological replicates of the experiment, including the one shown in Figure 6L (ANOVA: ***P ≤ 0.001).

**Figure S8- Related to Figure 7**

(A) Bar graph of average percent (± SEM) of deformed untreated or TGFβ-stimulated 8-day old 3D-organoids derived from NMuMG cells transfected as in Figure 7B from three biological replicates of the experiment, including the one shown in Figure 7C (ANOVA: ***P ≤ 0.001). (B) Bar graph of average percent (± SEM) of deformed untreated or TGFβ-stimulated 8-day old 3D-organoids (± SEM) derived from NMuMG cells transfected as in Figure 7F from three biological replicates of the experiment, including the one shown in Figure 7G (ANOVA: **P ≤ 0.01, ***P ≤ 0.001).

**Figure S9- Related to Figure 8**

Bar graph of average percent (± SEM) of deformed untreated or TGFβ-stimulated 8-day old 3D-organoids derived from NMuMG cells transfected as in as in Figure 8B from five biological replicates of the experiment, including the one shown in Figure 8C (ANOVA: ***P ≤ 0.001).
