# Supplementary figures and images for: Regulation of epithelial-mesenchymal transition and organoid morphogenesis by a novel TGFβ-TCF7L2 isoform-specific signaling pathway
Source: Cell Death Dis. 2020 Aug 25;11(8):704. doi: 10.1038/s41419-020-02905-z (PMC7447769; doi:10.1038/s41419-020-02905-z)

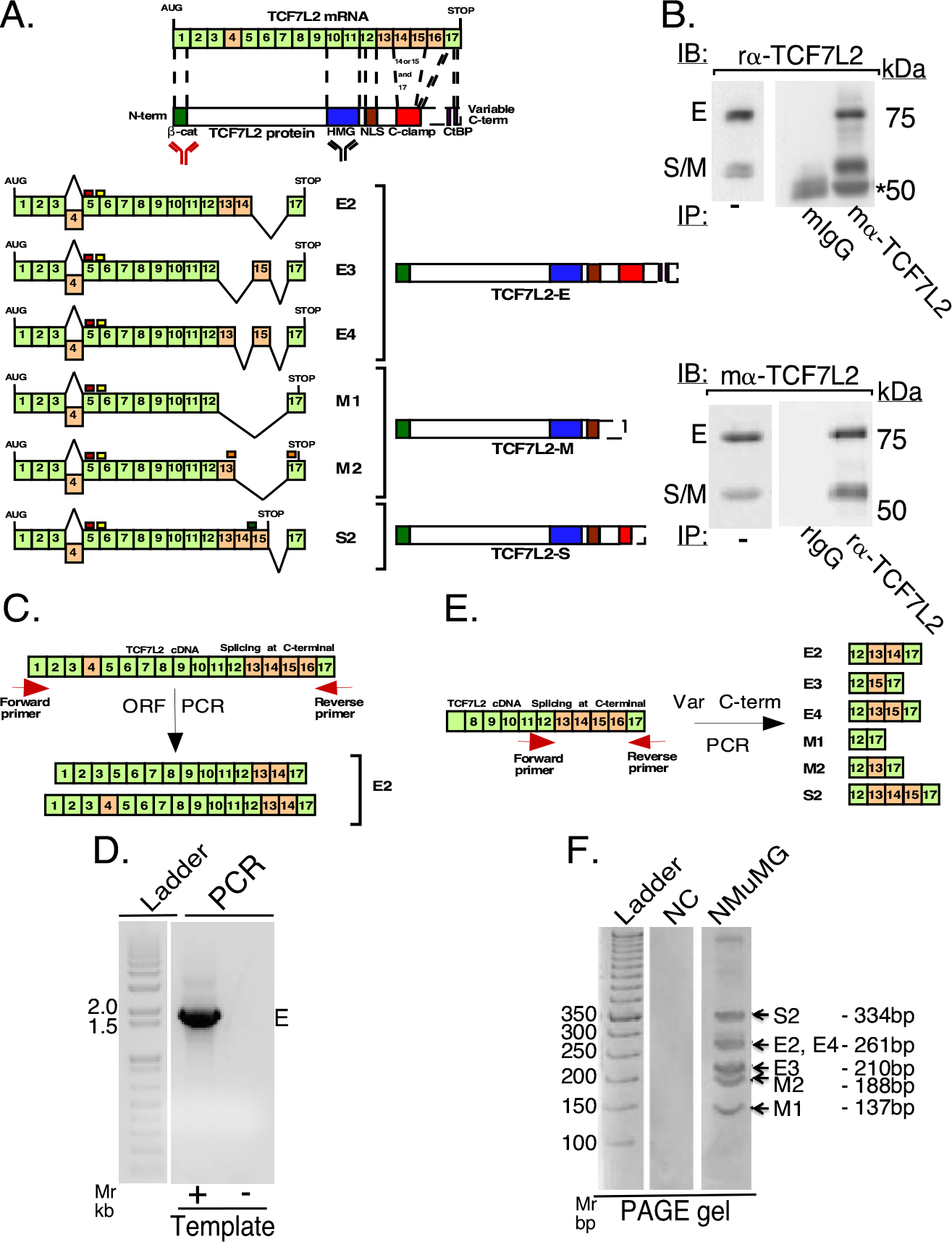

Supplement: Supplementary file 2 — Supplementary Figure 1 [file 41419_2020_2905_MOESM2_ESM.tif]

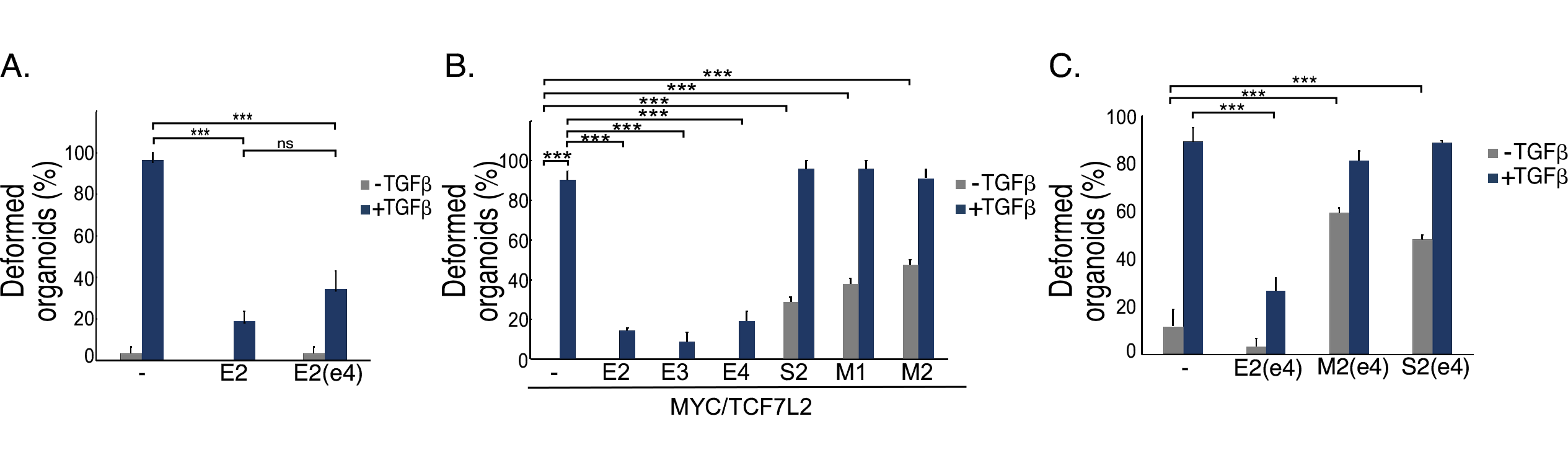

Supplement: Supplementary file 3 — Supplementary Figure 2 [file 41419_2020_2905_MOESM3_ESM.tif]

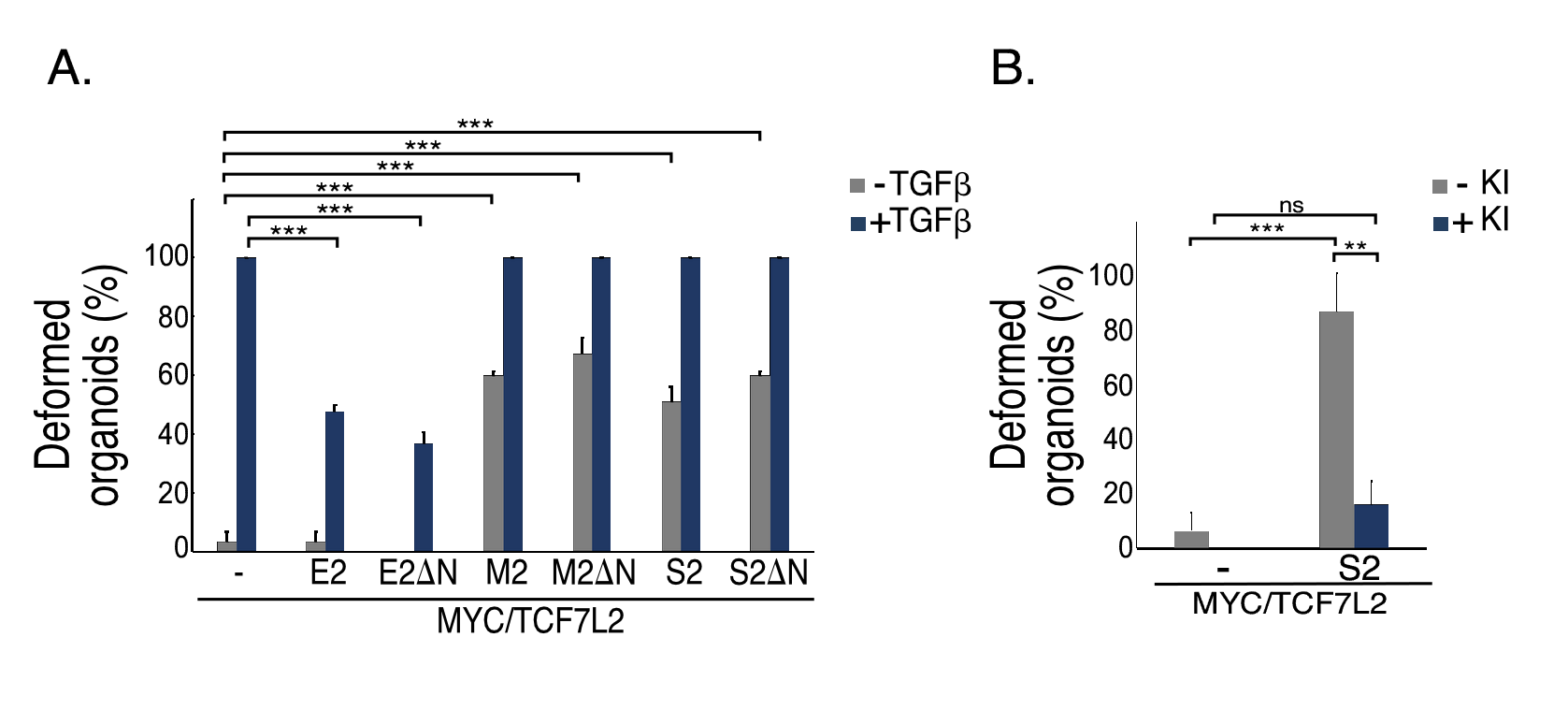

Supplement: Supplementary file 4 — Supplementary Figure 3 [file 41419_2020_2905_MOESM4_ESM.tif]

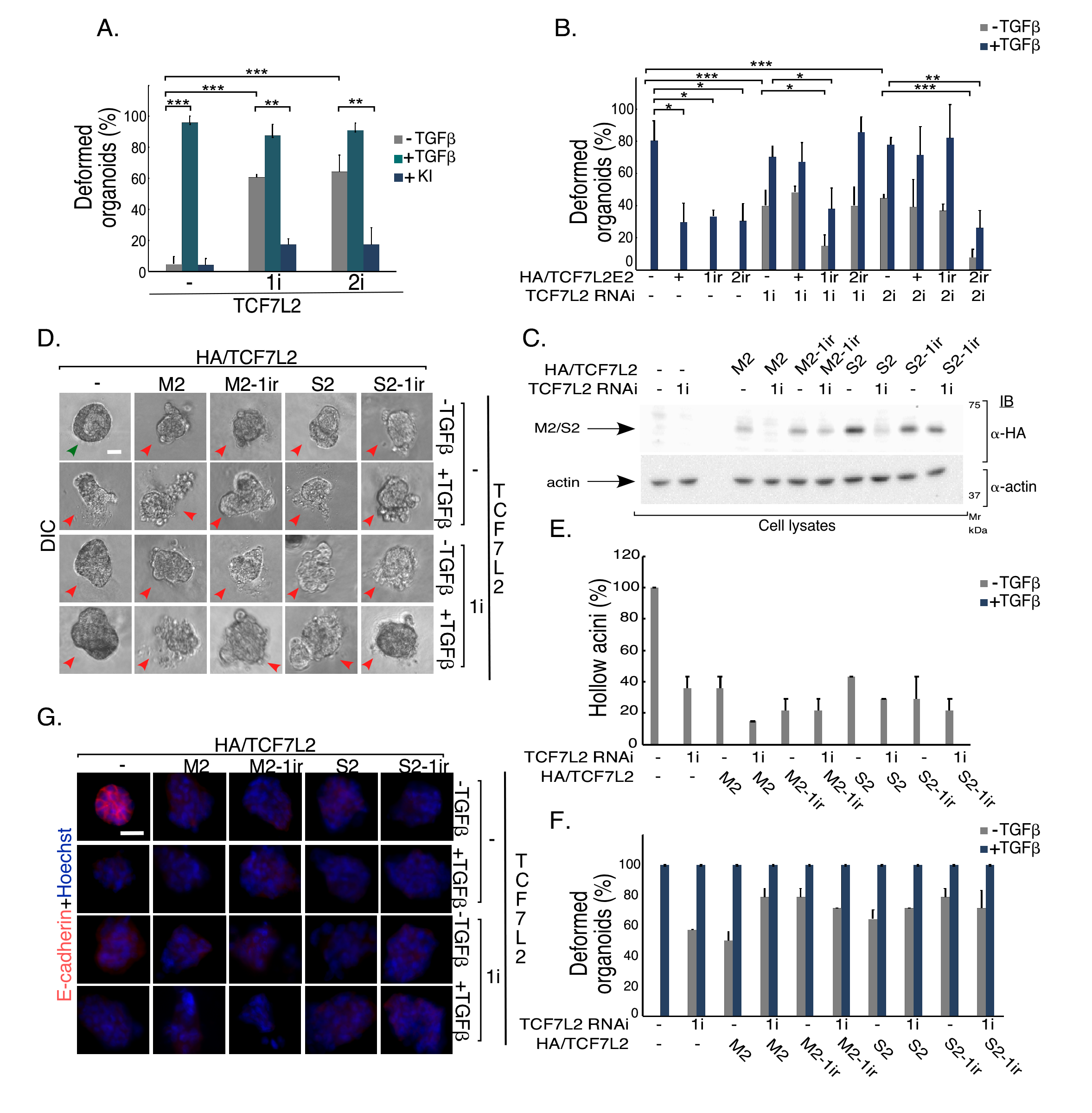

Supplement: Supplementary file 5 — Supplementary Figure 4 [file 41419_2020_2905_MOESM5_ESM.tif]

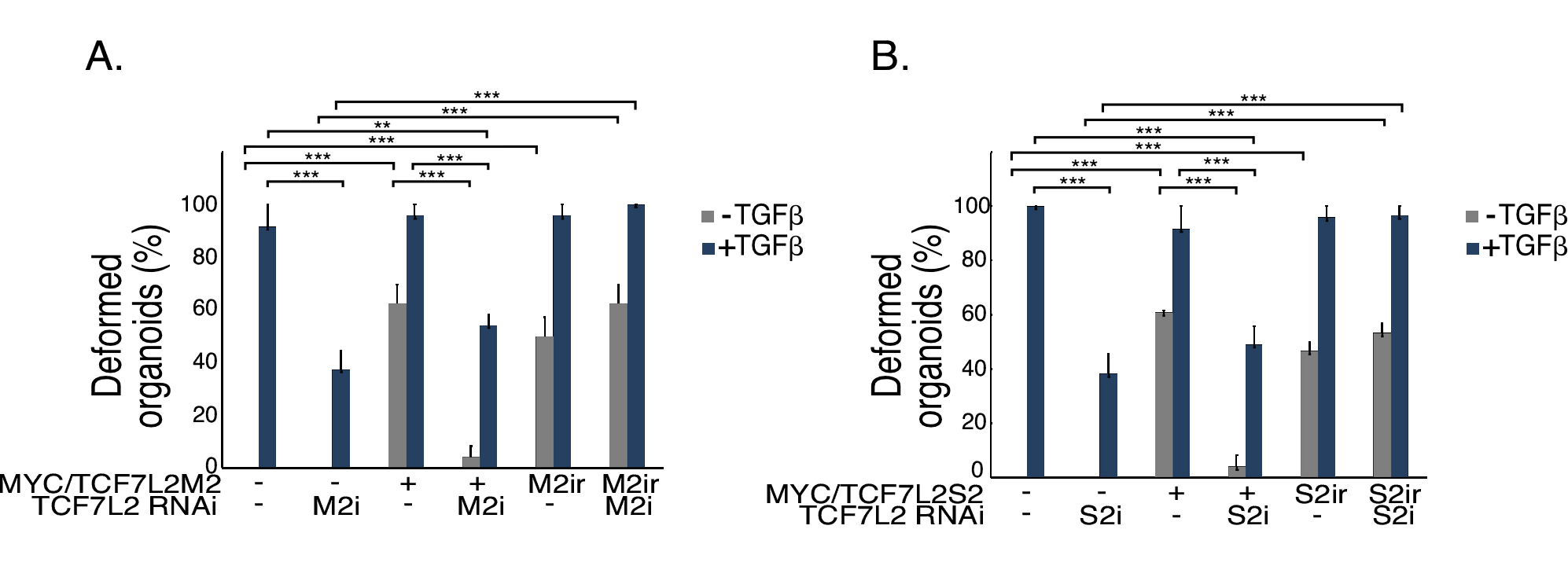

Supplement: Supplementary file 6 — Supplementary Figure 5 [file 41419_2020_2905_MOESM6_ESM.tif]

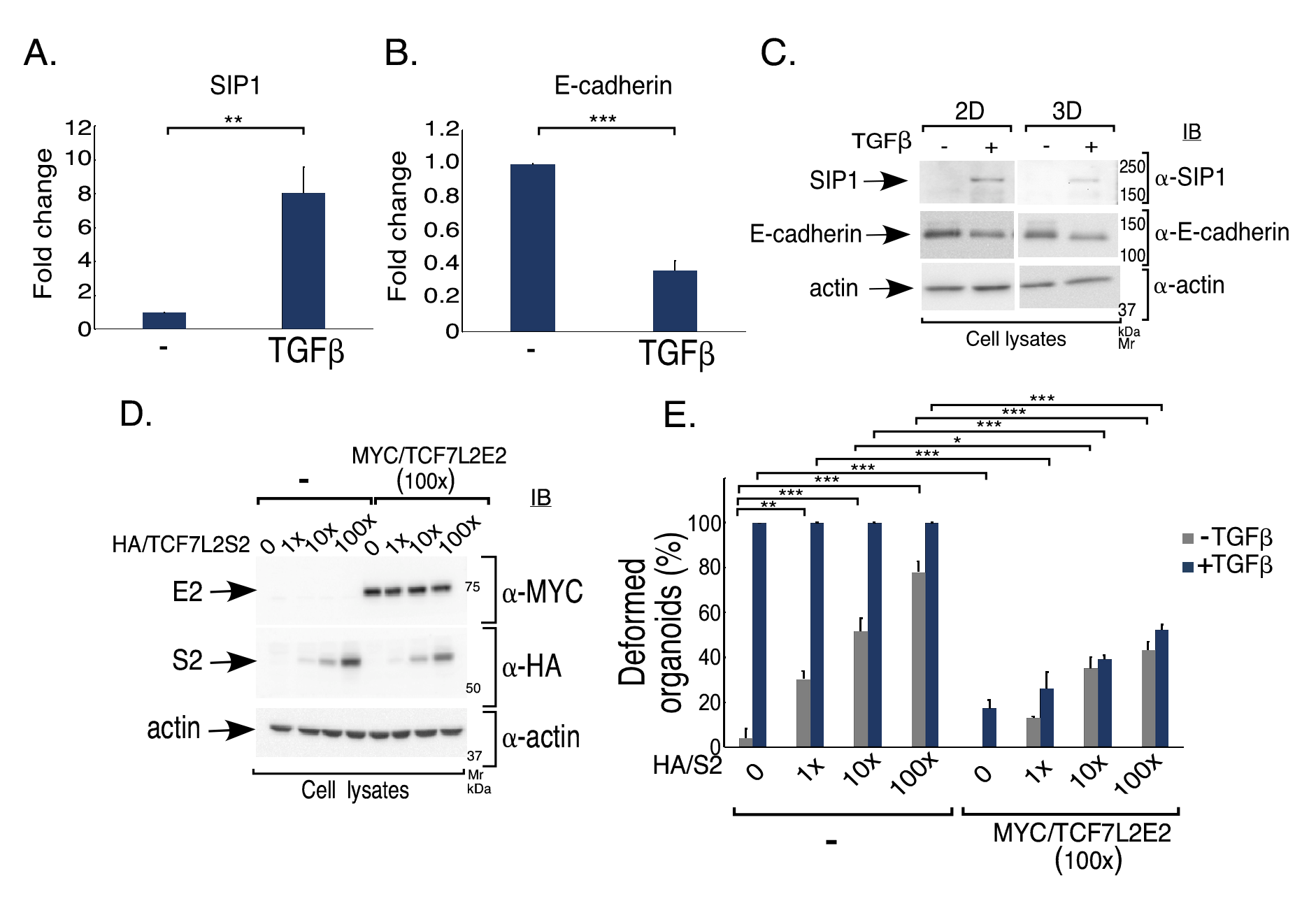

Supplement: Supplementary file 7 — Supplementary Figure 6 [file 41419_2020_2905_MOESM7_ESM.tif]

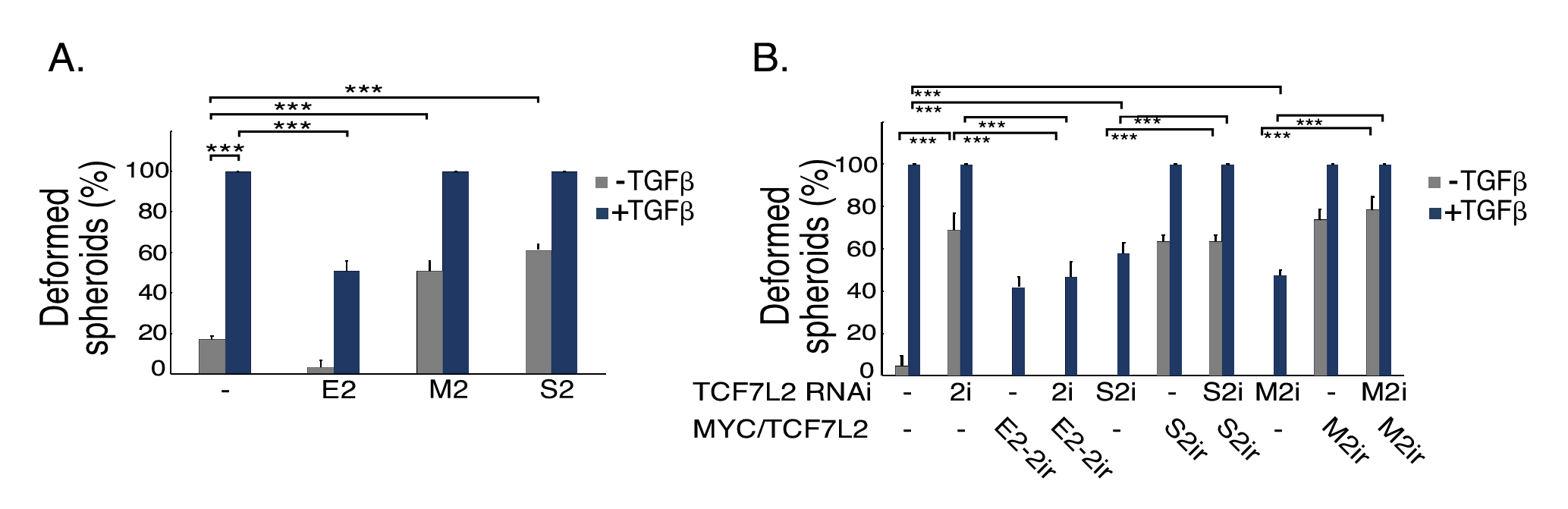

Supplement: Supplementary file 8 — Supplementary Figure 7 [file 41419_2020_2905_MOESM8_ESM.tif]

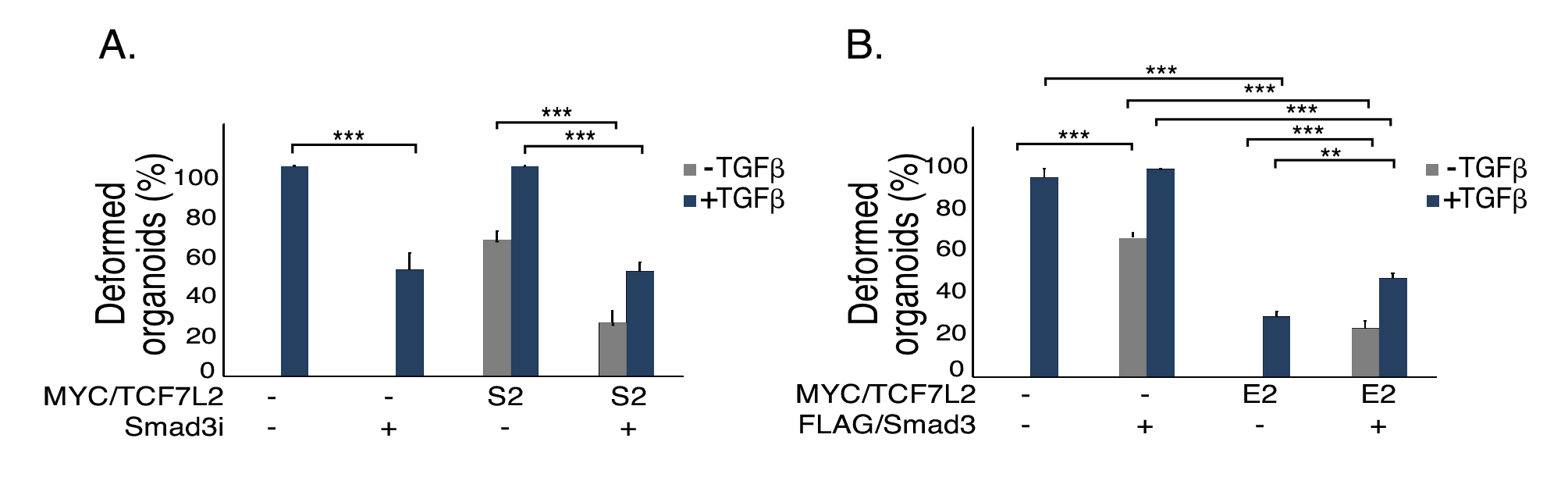

Supplement: Supplementary file 9 — Supplementary Figure 8 [file 41419_2020_2905_MOESM9_ESM.tif]

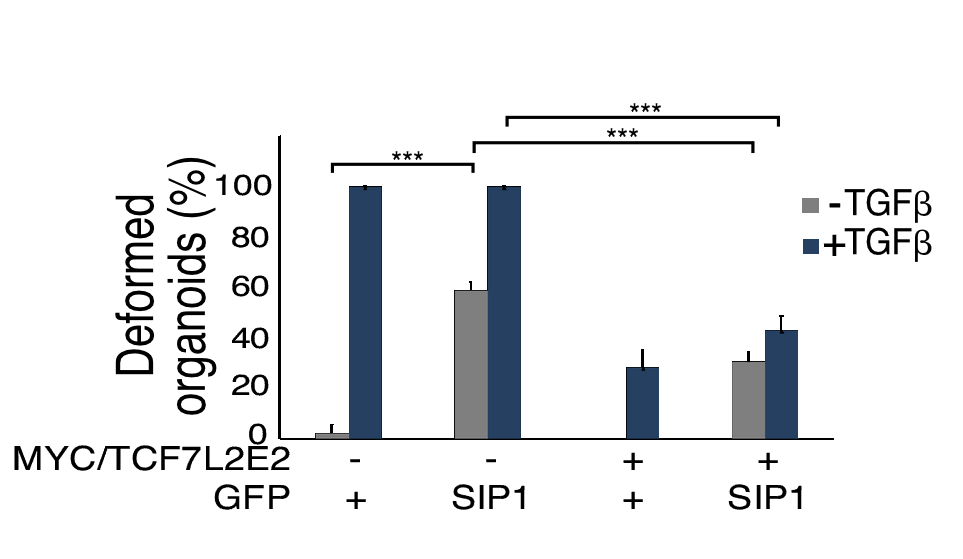

Supplement: Supplementary file 10 — Supplementary Figure 9 [file 41419_2020_2905_MOESM10_ESM.tif]
